# Synthesis and bioactivity of (13*Z*,15*E*)‐octadecadienal: A sex pheromone component from *Micromelalopha siversi* Staudinger (Lepidoptera: Notodontidae)

**DOI:** 10.1002/ps.6015

**Published:** 2020-08-31

**Authors:** Fu Liu, Li Guo, Sufang Zhang, Xiangbo Kong, Zhen Zhang

**Affiliations:** ^1^ Research Institute of Forest Ecology, Environment and Protection, Chinese Academy of Forestry Key Laboratory of Forest Protection of National Forestry and Grassland Administration Beijing China; ^2^ School of Biological Science and Engineering Xingtai University Xingtai China

**Keywords:** *Micromelalopha siversi*, sex pheromone, *Z*13,*E*15‐18:Ald, chemical synthesis

## Abstract

**BACKGROUND:**

*Micromelalopha siversi* (Staudinger) (Lepidoptera: Notodontidae) is a defoliator of poplar trees, *Populus* spp. (Salicaceae). In our previous study, 13,15‐octadecadienal has been conformed as a female‐produced candidate sex pheromone component of *M. siversi*, but the *Z*/*E* stereochemistry of the 1,3‐diene system has not been identified so far.

**RESULTS:**

Four unsaturated aliphatic aldehydes, *Z*13,*E*15‐18:Ald, *Z*13,*Z*15‐18:Ald, *E*13,*E*15‐18:Ald, and *E*13,*Z*15‐18:Ald, were synthesized from the commercially available 12‐bromo‐1‐decanol mainly by alkylation of lithium alkyne, normal Wittig or Wittig–Schlosser olefination, and hydroboration‐protonolysis. According to gas chromatography (GC) analysis of pheromone gland extracts, *Z*13,*E*15‐18:Ald was the main component, and a small amount of *Z*13,*Z*15‐18:Ald was also detected, with a ratio of approximately 7:3. However, the results of GC‐electroantennographic detection (GC‐EAD) showed that *Z*13,*E*15‐18:Ald was the only compound with electrophysiological activity, whereas *Z*13,*Z*15‐18:Ald elicited no activity. In the field, traps baited with only *Z*13,*E*15‐18:Ald resulted in much superior results to those with *Z*13,*Z*15‐18:Ald as well as the *Z*13,*E*15‐18:Ald and *Z*13,*Z*15‐18:Ald binary mixture.

**CONCLUSIONS:**

Based on geometrically selective synthesis and bioactivity tests, the active sex pheromone component of *M. siversi* has been identified as *Z*13,*E*15‐18:Ald, the pheromone component that has not been identified in Lepidoptera before. The synthetic component was attractive to male moths in preliminary field traps, which provides novel technologies to monitor and control this pest.

## Graphical abstract

The sex pheromone of *Micromelalopha siversi* was characterized as (13*Z*,15*E*)‐octadecadienal, the pheromone component that has not been identified in Lepidoptera before. The total synthesis of this active component was also reported.

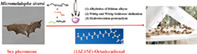

© 2020 The Authors. *Pest Management Science* published by John Wiley & Sons Ltd on behalf of Society of Chemical Industry.

## 1 INTRODUCTION

Poplar (*Populus* sp.) represents the most widely distributed and adaptable tree species in the world. An increasing amount of land is being used to plant poplars, particularly in China, South Korea, and the USA. Meanwhile, many countries with limited natural forests use poplars from plantations as an important timber source.^1^ In addition, poplar plantations are also being investigated as renewable sources of energy for environmental improvement,^2^, ^3^, ^4^ especially for important short rotation coppice (SRC) plantations, which contain and absorb vast quantities of atmospheric carbon dioxide.^5^, ^6^, ^7^, ^8^

*Micromelalopha siversi* (Staudinger) (Lepidoptera: Notodontidae), which is mainly distributed in China, is one of the defoliators that severely damage poplar plantations.^9^ In addition, *M. siversi* larvae usually injure the mesophyll, causing balding of poplar branches, weakening the host and curtailing growth. Over recent decades, the biological characteristics of *M. siversi* have been extensively studied.^10^, ^11^ Commonly, *M. siversi* has three to four generations in northeastern China, and five to seven generations in south‐central China, but the generations overlap extensively. The females have a clear circadian rhythm‐related calling behavior during the scotophase, but not during the light period.^12^ After mating, females will deposit their eggs on the poplar leaves. The larvae have five instar stages, and the mature larvae will pupate in the deciduous layer overwinter.^13^

Outbreaks of this leaf‐feeding pest across China have resulted in the wide application of natural insecticides or artificially synthesized pyrethroids, negatively affecting biodiversity as well as natural enemies within the ecosystem.^14^, ^15^ Therefore, more environmentally acceptable approaches are required to control *M. siversi* effectively.

At present, mass trapping or mating disruption using species‐specific sex pheromone traps are effective approaches to control numerous Lepidoptera species.^16^, ^17^ The sex pheromone component plays an important role in contacting and promoting the chemical communication and reproductive behavior of *M. siversi*. 13,15‐octadecadienal, an unsaturated aliphatic aldehyde, was found to be the sex pheromone component of *M. siversi* by our group in 2019.^18^ However, the stereochemistry concerning the double bond at C‐13 and C‐15 has not yet been characterized.

In this study, the total synthesis of *Z*13,*E*15‐18:Ald, *Z*13,*Z*15‐18:Ald, *E*13,*E*15‐18:Ald, and *E*13,*Z*15‐18:Ald is presented. Moreover, electrophysiological and behavior tests were carried out to characterize the active sex pheromone component of *M. siversi* and to prepare an effective lure to monitor and control *M. siversi*.

## 2 MATERIALS AND METHODS

### 2.1 Insects and pheromone extraction

*M. siversi* pupae were obtained from Suiping (Henan Province, China), separated by sex and reared under the following conditions, temperature of 26 ± 1 °C, light/dark cycle of 14 h/10 h, together with relative humidity (RH) of 70 ± 5%. The adults were raised with 10% honey solution that was put onto cotton. On days 1 or 2 following emergence, the female moths were adopted to extract pheromone, whereas the male counterparts were utilized in gas chromatography‐electroantennographic detection (GC‐EAD) analysis.

For the virgin calling female moths, their abdominal tips were cut, followed by extraction with distilled hexane for 30–40 min. Thereafter, the resultant hexane extracts were placed in 2‐mL glass vials (Agilent Technologies, Palo Alto, CA, USA) and preserved in a refrigerator (Haier, Qingdao, Shandong, China) at −20 °C for subsequent chemical analyses.

### 2.2 GC‐EAD analysis

An Agilent 7890A GC containing a flame ionization detector (FID) was used to perform coupled GC‐EAD. A Y splitter (5181–3397, Agilent Technologies) along with an HP‐5 capillary column (inside diameter 30 m × 250 μm, thickness of film 0.25 μm; Agilent Technologies) was used in analyses. As the effluent of the GC column, the carrier gas (hydrogen and nitrogen) was separated at a ratio of 1:1 to simultaneously detect between the FID and the EAD apparatuses. A 1‐μL splitless sample was added at 220 °C (inlet temperature). The oven was held at 60 °C for 2 min, then programmed to 250 °C at a heating rate of 8 °C min^−1^. This final temperature was maintained for 10 min. An EAD probe with high resistance, an Intelligent Data Acquisition Controller (CS‐55), type IDAC‐02, along with a Signal Interface Box (Syntech, Buchenbach, Germany) were used to detect antennal depolarization. The basic segment of the moth at 1–2 days of age was cut with caution to prepare freshly resected male antenna, which was later added to a glass capillary filled with 0.9% normal saline housing 0.39‐mm silver wires (Sigmund Cohn Corp, Mt. Vernon, NY, USA). Electrodes were connected to a combi Probe (PRG‐3, Syntech). The humid air filtered with charcoal flowed through the glass tube at a flow rate of 1 L min^−1^. The glass tube was connected to the GC transfer line, which was designed to track the temperature of GC oven. A compound that was able to elicit an antennal response at least five times was considered to show electroantennographical activity.

### 2.3 Gas chromatography–mass spectrometry analysis

The chemicals analysis was carried out using an Agilent GC coupled with a mass spectrometry system (TRACE GC 2000). The GC system was equipped with a DB‐5 ms column (30 m × 0.25 mm × 0.25 μm). Samples of 1 μL from different solutions were injected manually into the system at an injector temperature of 230 °C under the splitless mode. The oven was held for 2 min at 80 °C, then heated to 190 °C at a heating rate of 15 °C min^−1^, and maintained at thei temperature for 10 min. The carrier gas (helium) was injected at a flow rate of 1.2 mL min^−1^, filament bias voltage 70 eV, and ion source temperature of 250 °C. The scanning mode was adopted to obtain spectra (range of mass *m/z* 35–500). Compound retention times (RTs) were compared with synthesized standards to identify the compounds. The NIST11 library (Scientific Instrument Services, Inc., Ringoes, NJ, USA) was used to obtain mass spectra for reference.

### 2.4 Gas chromatography coupled with flame ionization detection

GC‐FID analysis was performed on an Agilent 7890A equipped with a 30 m × 0.25 mm × 0.25‐μm HP‐FFAP column (Agilent Technologies). Samples of 1 μL from different solutions were injected manually into the system at the split mode (ratio 1:40) with an injector temperature of 220 °C. The oven was held for 2 min at 100 °C, then heated to 190 °C at a heating rate of 15 °C min^−1^, maintained for 10 min, then further heated to 225 °C at a heating rate of 8 °C min^−1^, and maintained for 10 min. Carrier gas (nitrogen) was injected at a flow rate of 1.0 mL min^−1^. An FID operating at 230 °C was used for detection. The pheromone components were quantified relative to the external standard (1 μL aliquot of 5 ng μL^−1^ *n*‐tridecane).

### 2.5 Chemicals

All reactants used for synthesizing the four diastereomers of 13,15‐octadecadienal were purchased from Sigma‐Aldrich (St Louis, MO, USA). The solvents used to prepare gland extracts and to carry out chromatographic analyses were at HPLC grade and provided by Sigma‐Aldrich, while those used in synthesizing compounds were at Pro analysis grade and provided by Aladdin (Shanghai, China). A Bruker NMR spectrometer (Bruker, Fällanden, Switzerland) was used to record the NMR spectra in CDCl_3_ (^1^H and ^13^C at 500 and 125 MHz, respectively), with tetramethylsilane as the internal standard.

### 2.6 Synthesis

**Scheme 1:**
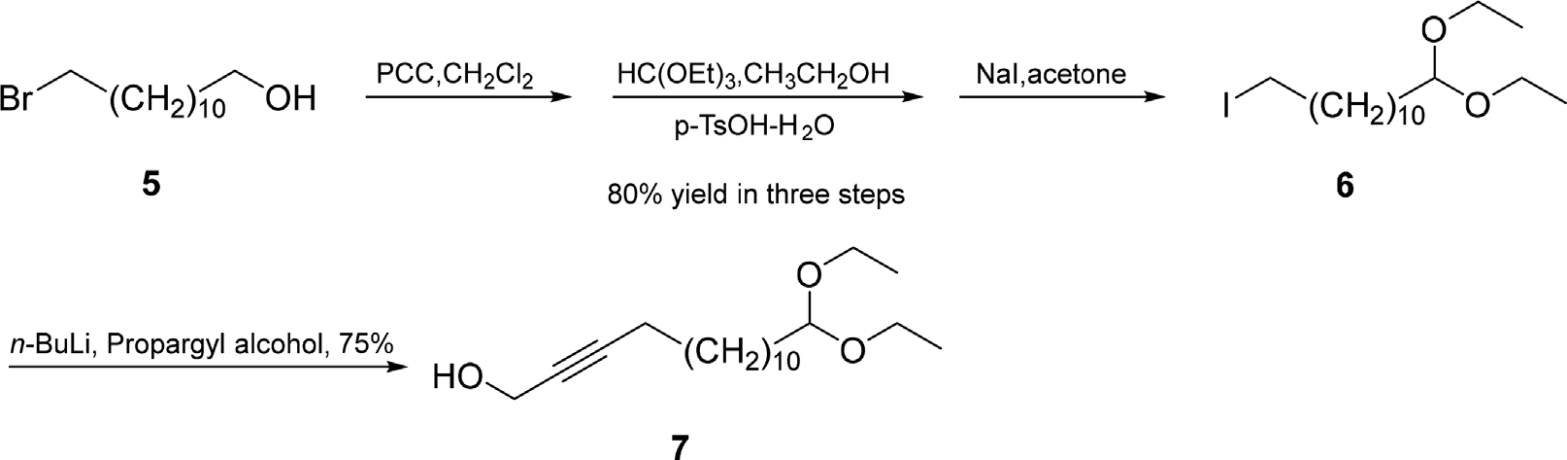
The synthetic route of 15,15‐diethoxypentadec‐2‐yn‐1‐ol **7**. PCC, pyrindium chlorochromate; HC(OEt)_3_, triethoxy methane; *p*‐TsOH‐H_2_O, *p*‐toluenesulfonic acid monohydrate; NaI, sodium iodide.

**Scheme 2:**
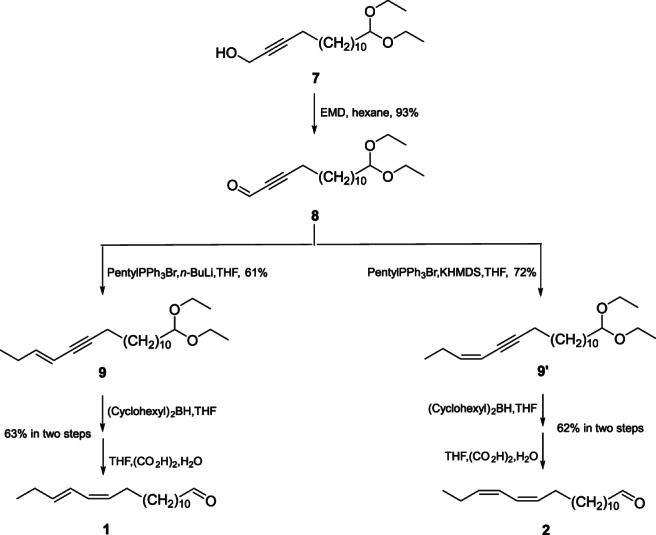
The synthetic routes of the (13*Z*,15*E*)‐ and (13*Z*,15*Z*)‐octadecadienal isomers. EMD, electrolytic manganese dioxide; PentylPPh_3_Br, pentyl(triphenyl)phosphanium bromide; *n*‐BuLi, *n*‐butyllithium; THF, tetrahydrofuran; KHMDS, potassium *bis*(trimethylsilyl)amide; (Cyclohexyl)_2_BH, dicyclohexylborane; (CO_2_H)_2_, oxalic acid.

**Scheme 3:**
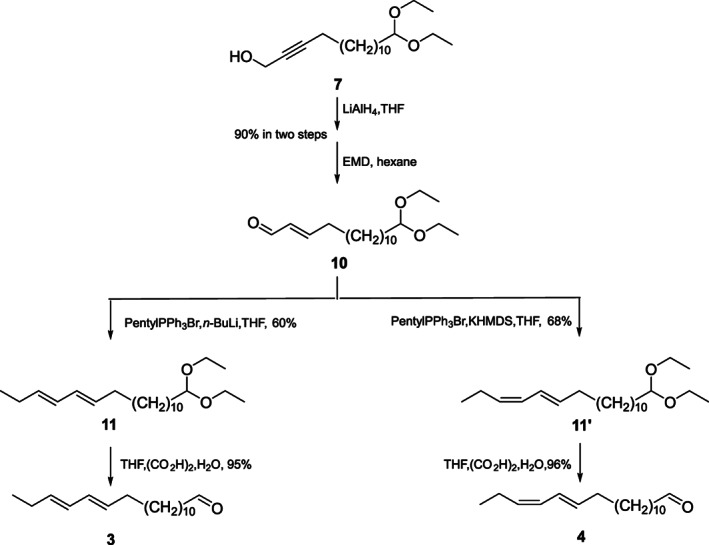
The synthetic routes of the (13*E*,15*E*)‐ and (13*E*,15*Z*)‐octadecadienal isomers. LiAlH_4_, lithium aluminium hydride; EMD, electrolytic manganese dioxide; PentylPPh_3_Br, pentyl(triphenyl)phosphanium bromide; *n*‐BuLi, *n*‐butyllithium; THF, tetrahydrofuran; KHMDS, potassium *bis*(trimethylsilyl)amide; (Cyclohexyl)_2_BH, dicyclohexylborane; (CO_2_H)_2_, oxalic acid.

The general synthetic procedures for diastereomers of 13,15‐octadecadienal (**1**–**4**) were as follows (Schemes 1, 2, 3).

#### 2.6.1 *1,1‐Diethoxy‐12‐iododecane (* ***6*** *)*

Pyridinium chlorochromate (86.0 g, 400 mmol) was used to oxidize 12‐bromodecanol **5** (52.8 g, 200 mmol) in CH_2_Cl_2_ (800 mL) under ambient temperature for 4 h. The solvent removal was conducted at reduced pressure, while petroleum was used to wash the residual. Typically, solvent removal in vacuum resulted in a dark oil. SiO_2_ was used to chromatograph the residue, while the crude 10‐bromodecanal was produced through eluting using hexane:ethyl acetate (50:1, v/v) (Scheme 1).

Triethyl orthoformate (44.5 g, 300 mmol) was mixed with *p*‐sulphonic acid monohydrate (0.57 g, 3 mmol) and the resultant mixture was placed into 300 mL of anhydrous ethanol solution containing crude 10‐bromodecanal at 0 °C. Later, the resultant mixture was allowed to stand overnight at 0 °C. Next, water and K_2_CO_3_ solution were added to the mixture to prepare the basic solution. Diethyl ether was used to extract the mixture, followed by brine washing, MgSO_4_ drying and solvent removal at reduced pressure. Finally, chromatographic analysis of the obtained residue was conducted on silica with hexane and ethyl acetate (30:1, v/v), resulting in crude 1,l‐diethoxy‐12‐bromodecanal being obtained.

Later, the as‐obtained product was stirred using sodium iodide (90 g, 600 mmol) within dry acetone (500 ml) in the presence of reflux to convert to iodide. After solvent removal at reduced pressure, water (200 mL) was used to dilute the mixture, while petroleum was used to extract the product. The extracts were rinsed with water, brine and 1% Na_2_S_2_O_3_ solution, followed by Na_2_SO_4_ drying, concentration at reduced pressure, and chromatographic analysis of the residue on SiO_2_. After eluting using hexane:ethyl acetate (30:1, v/v) a colorless oil was obtained, which was 1,1‐diethoxy‐12‐iododecan (**6**) in 80% yield (61.4 g) according to 12‐bromodecanol. ^1^H NMR (500 MHz, CDCl_3_, ppm) *δ*: 1.19 (6H, t, *J* = 7.0 Hz, 2CH_3_), 1.29 (16H, m, 8CH_2_), 1.60 (2H, m, H‐2), 1.82 (2H, m, H‐11), 3.18 (2H, t, *J* = 7.0 Hz, H‐12), 3.48 (2H, m, CH_2_O), 3.64 (2H, m, CH_2_O), 4.47 (1H, t, *J* = 6.0 Hz, OCHO). ^13^C NMR (125 MHz, CDCl_3_, ppm) *δ*: 102.9 (OCHO), 60.8 (CH_2_O), 60.8 (CH_2_O), 33.6 (C‐2), 33.5 (C‐11), 30.5 (C‐10), 29.5 (CH_2_), 29.5 (CH_2_), 29.5 (CH_2_), 29.4 (CH_2_), 29.4 (CH_2_), 28.5 (CH_2_), 24.7 (CH_2_), 15.3 (CH_3_), 15.3 (CH_3_), 7.3 (C‐12).

#### 2.6.2 *15,15‐Diethoxypentadec‐2‐yn‐1‐ol (* ***7*** *)*

*n*‐Butyllithium (2.5 M in hexane) (160 mL, 400 mol) was slowly added to a solution of propargyl alcohol (11.2 g, 200 mol) in hexamethyl phosphoryl triamide (HMPT) and tetrahydrofuran (THF; 800 mL, 1:1, v/v) at −40 °C in the presence of argon. After stirring for 30 min, 1,1‐diethoxy‐12‐iododecane (**6**; 38.4 g, 100 mmol) in HMPT:THF mixed solution (50 mL, 1:1, v/v) was added over 20 min, followed by overnight stirring. Later, water was added to quench the reaction mixture, while ethyl acetate was added for extraction. Afterwards, water was added to wash the organic layer, then Na_2_SO_4_ was used for drying and the crude product was obtained after evaporation. The colorless oil 15,15‐diethoxypentadec‐2‐yn‐1‐ol (**7**) was obtained (23.4 g, 75% yield) after elution with hexane:ethyl acetate (10:1, v/v). ^1^H NMR (500 MHz, CDCl_3_, ppm) *δ*: 1.19 (6H, t, *J* = 7.0 Hz, 2CH_3_), 1.25–1.31 (16H, m, 8CH_2_), 1.46–1.52 (2H, m, H‐11), 1.57–1.61 (2H, m, H‐2), 2.12–2.21 (2H, m, H‐12), 3.45–3.51 (2H, m, CH_2_O), 3.60–3.66 (2H, m, CH_2_O), 4.24 (2H, t, *J* = 2.0 Hz, H‐15); 4.47 (1H, t, *J* = 6.0 Hz, OCHO). ^13^C NMR (125 MHz, CDCl_3_, ppm) *δ*: 102.9 (OCHO), 86.5 (C‐13), 78.2 (C‐14), 60.8 (CH_2_O), 60.8 (CH_2_O), 51.3 (C‐15), 33.5 (C‐2), 29.5 (CH_2_), 29.5 (CH_2_), 29.5 (CH_2_), 29.4 (CH_2_), 29.4 (CH_2_), 29.0 (CH_2_), 28.8 (CH_2_), 28.6 (CH_2_), 24.7 (C‐3), 18.7 (C‐12), 15.3 (CH_3_), 15.3 (CH_3_).

#### 2.6.3 *15,15‐Diethoxypentadec‐2‐ynal (* ***8*** *)*

Electrolytic manganese dioxide (EMD; 52.1 g, 600 mmol) was placed into a dry hexane solution (300 mL) of 15,15‐diethoxypentadec‐2‐yn‐1‐ol (**7**; 10.0 g, 32.0 mmol). The resultant solution was subjected to 4 h of stirring under ambient temperature, then the manganese‐containing residues were filtered off. The protected aldehyde **8** (a pale‐yellow oil) was obtained after elution with hexane:ethyl acetate (20:1, v/v), resulting in a 93% yield (9.24 g). ^1^H NMR (500 MHz, CDCl_3_, ppm) *δ*: 1.20 (6H, t, *J* = 7.0 Hz, 2CH_3_), 1.26–1.33 (16H, m, 8CH_2_), 1.38–1.42 (2H, m, H‐11), 1.57–1.60 (2H, m, H‐2), 2.40 (2H, t, *J* = 7.5 Hz, H‐12), 3.45–3.53 (2H, m, CH_2_O), 3.60–3.66 (2H, m, CH_2_O), 4.47 (1H, t, *J* = 6.0 Hz, OCHO), 9.17 (1H, s, CHO). ^13^C NMR (125 MHz, CDCl_3_, ppm) *δ*: 177.3 (CHO), 102.9 (OCHO), 94.5 (C‐13), 77.2 (C‐14), 60.8 (OCH_2_O), 60.8 (OCH_2_O), 33.6 (C‐2), 29.5 (CH_2_), 29.5 (CH_2_), 29.5 (CH_2_), 29.4 (CH_2_), 29.3 (CH_2_), 28.8 (CH_2_), 28.7 (CH_2_), 27.5 (CH_2_), 24.7 (C‐3), 19.1 (C‐12), 15.3 (CH_3_), 15.3 (CH_3_).

#### 2.6.4 *(*E*)‐18,18‐Diethoxyoctadec‐3‐en‐5‐yne (* ***9*** *)*

Propyl triphenylphosphonium bromide (4.62 g, 12.0 mmol) was suspended in THF (100 mL), followed by 30 min of stirring with *n*‐BuLi (4.8 mL, 2.5 M in hexane). The resulting mixture was cooled to −70 °C, followed by the addition of aldehyde **8** (3.1 g, 10.0 mmol) in THF (5 mL). The mixture was stirred vigorously until the yellow coloration disappeared, and an additional amount of *n*‐BuLi (2.5 M, 4.8 mL, 12.0 mmol) was added. The reaction mixture was stirred at −30 °C for 5 min, whereupon a solution of hydrogen chloride in ethyl ether (1 M, 13.0 mmol, 13.0 mL) and then potassium tertbutoxide (2.04 g, 18.2 mmol) in 2‐methyl‐2‐propanol (1.35 g, 18.2 mmol) were added. Later, the mixed solution was subjected to 2 h of stirring under ambient temperature, water washing till neutrality, and MgSO_4_ drying. After evaporation of the solvent, the crude product (*E*‐**9**:*Z*‐**9′** = 7:1) was subjected to 10% silver nitrate SiO_2_ with hexane:ethyl acetate (60:1, v/v), yielding 61% (2.65 g) of the *E*‐**9** as a colorless oil. ^1^H NMR (500 MHz, CDCl_3_, ppm) *δ*: 0.99 (3H, t, *J* = 7.5 Hz, CH_3_), 1.19 (6H, t, *J* = 7.0 Hz, 2CH_3_), 1.26–1.28 (16H, m, 8CH_2_), 1.47–1.52 (2H, m, H‐11), 1.57–1.61 (2H, m, H‐3), 2.06–2.12 (2H, m, H‐17), 2.25–2.29 (2H, m, H‐12), 3.45–3.51 (2H, m, OCH_2_), 3.60–3.66 (2H, m, OCH_2_), 4.47 (1H, t, *J* = 6.0 Hz, OCHO), 5.44 (1H, m, H‐15), 6.07 (1H, dt, *J* = 13.5, 6.5 Hz, H‐16). ^13^C NMR (125 MHz, CDCl_3_, ppm) *δ*: 144.6 (C‐16), 108.9 (OCHO), 102.9 (C‐15), 88.8 (C‐13), 79.1 (C‐14), 60.8 (CH_2_‐O), 60.8 (CH_2_‐O), 33.6 (C‐2), 29.6 (CH_2_), 29.5 (CH_2_), 29.5 (CH_2_), 29.5 (CH_2_), 29.5 (CH_2_), 29.1 (CH_2_), 28.9 (CH_2_), 28.8 (CH_2_), 26.0 (C‐17), 24.7 (C‐3), 19.3 (C‐12), 15.3 (CH_3_), 15.3 (CH_3_), 13.4 (C‐18).

#### 2.6.5 *(13*Z*,15*E*)‐Octadecadienal (* ***1*** *)*

A solution of compound **9** (2.0 g, 5.95 mmol) in THF (10 mL) was added dropwise to dicyclohexylborane solution (12.0 mmol). The suspension was stirred at −15 °C for 2 h, naturally heating to ambient temperature and further stirred under ambient temperature for 2 h until no dicyclohexylborane precipitate was observed. The resulting solution was mixed with 2 mL of glacial acetic acid and stirred at 50 °C for 2 h. Subsequently, 3 mL of 6 M sodium hydroxide and 3 mL of 35% hydrogen peroxide were added in succession to oxidize the resultant dicyclohexylborinate. The mixture was stirred for an additional 30 min and poured into 15 mL of ice water, extracted with hexane and dried with MgSO_4_. After evaporation, the crude product was added to oxalic acid dihydrate (3.0 g) in a solution of THF and water (60 mL, 1:1, v/v), followed by stirring and heating of the obtained mixture under 60 °C in the presence of argon for 40 min. Afterwards, hexane was used to extract the mixture. Water, brine and sodium bicarbonate solution were used to wash organic solution, followed by Na_2_SO_4_ drying and vacuum concentration. Column chromatography at medium pressure (hexane:ethyl acetate = 50:1, v/v) was conducted to analyze the residue, and the colorless oil **1** was produced (0.99 g, 63% yield, based on **9**, isomeric purity of >95%). ^1^H NMR (500 MHz, CDCl_3_, ppm) *δ*: 1.01 (3H, t, *J* = 7.5 Hz, CH_3_), 1.26–1.30 (16H, m, 8CH_2_), 1.60–1.64 (2H, m, H‐3), 2.10–2.16 (4H, m, H‐12 and H‐17), 2.41 (2H, td, *J* = 7.5, 1.5 Hz, H‐2), 5.30 (1H, m, H‐13), 5.69 (1H, dt, *J* = 13.5, 7.0 Hz, H‐16), 6.59 (1H, t, *J* = 11.0 Hz, H‐14), 6.30 (1H, m, H‐15), 9.76 (1H, t, *J* = 2.0 Hz, CHO). ^13^C NMR (125 MHz, CDCl_3_, ppm) *δ*: 202.9 (CHO), 136.1 (C‐16), 130.2 (C‐13), 128.6 (C‐14), 124.7 (C‐15), 43.9 (C‐2), 29.7 (CH_2_), 29.6 (CH_2_), 29.6 (CH_2_), 29.5 (CH_2_), 29.4 (CH_2_), 29.4 (CH_2_), 29.3 (CH_2_), 29.2 (CH_2_), 27.7 (CH_2_), 25.9 (C‐3), 22.1 (C‐17), 13.7 (CH_3_). GC‐MS (70 eV, *m/z*): 264, 235, 221, 207, 147, 135, 121, 109, 95, 81, 67, 55.

#### 2.6.6 *(*Z*)‐18,18‐Diethoxyoctadec‐3‐en‐5‐yne (* ***9*** *′)*

A solution of potassium bis(trimethylsilyl)amide (0.5 M in toluene, 62 mL, 31.0 mmol) was added to *n*‐propyl triphenylphosphonium bromide (9.93 g, 25.8 mmol) in THF (150 mL). The resultant mixed solution was subjected to 1 h of reflux and cooled to −70 °C, then a solution of the aldehyde **8** (4.0 g, 12.9 mmol) in THF (20 mL) was added dropwise. The mixture was stirred for 3 h and added to 30 mL of 10% aqueous NH_4_Cl. After separating the organic phase, hexane was used to extract the aqueous phase and Na_2_SO_4_ was applied to dry the integrated organic phases. The crude product was obtained through evaporation and analyzed by column chromatography at medium pressure (hexane:ethyl acetate 60:1, v/v), and a *Z*‐**9′** and *E*‐**9** mixture (16:1, colorless oil) was obtained (3.12 g, 72% yield). ^1^H NMR (500 MHz, CDCl_3_, ppm) *δ*: 1.00 (3H, t, *J* = 7.5 Hz, CH_3_), 1.20 (6H, t, *J* = 7.0 Hz, 2CH_3_), 1.26–1.32 (16H, m, 8CH_2_), 1.50–1.54 (2H, m, H‐11), 1.57–1.62 (2H, m, H‐3), 2.27–2.34 (4H, m, H‐12 and H‐17), 3.47–3.50 (2H, m, OCH_2_), 3.61–3.65 (2H, m, OCH_2_), 4.47 (1H, t, *J* = 6.0 Hz, OCHO), 5.30 (1H, m, H‐15), 5.79 (1H, dt, *J* = 10.5, 7.0 Hz, H‐16). ^13^C NMR (125 MHz, CDCl_3_, ppm) *δ*: 144.0 (C‐16), 108.7 (OCHO), 102.9 (C‐15), 94.5 (C‐13), 77.2 (C‐14), 60.8 (CH_2_‐O), 60.8 (CH_2_‐O), 33.6 (C‐2), 29.5 (CH_2_), 29.5 (CH_2_), 29.5 (CH_2_), 29.5 (CH_2_), 29.5 (CH_2_), 29.1 (CH_2_), 28.9 (CH_2_), 28.8 (CH_2_), 24.7 (C‐17), 23.4 (C‐3), 19.5 (C‐12), 15.3 (CH_3_), 15.3 (CH_3_), 13.4 (C‐18).

#### 2.6.7 *(13*Z*,15*Z*)‐Octadecadienal (* ***2*** *)*

Compound **2** was prepared from compound **9′** (2.16 g), as described for preparing compound **1** based on compound **9**, at an isomeric purity of >96% and a 62% yield based on **9′** (1.02 g, colorless oil). ^1^H NMR (500 MHz, CDCl_3_, ppm) *δ*: 0.99 (3H, t, *J* = 7.5 Hz, CH_3_), 1.26 (16H, m, 8CH_2_), 1.59–1.63 (2H, m, H‐3), 2.15–2.20 (4H, m, H‐12 and H‐17), 2.41 (2H, td, *J* = 7.5, 1.5 Hz, H‐2), 5.44 (2H, m, H‐13 and 16), 6.12 (2H, m, H‐14 and 15), 9.75 (1H, t, *J* = 2.0 Hz, CHO). ^13^C NMR (125 MHz, CDCl_3_, ppm) *δ*: 202.9 (CHO), 133.6 (C‐16), 132.1 (C‐13), 123.4 (C‐14), 123.0 (C‐15), 43.9 (C‐2), 29.6 (C‐12), 29.5 (CH_2_), 29.5 (CH_2_), 29.5 (CH_2_), 29.4 (CH_2_), 29.3 (CH_2_), 29.3 (CH_2_), 29.1 (CH_2_), 27.4 (CH_2_), 22.1 (C‐3), 20.8 (C‐17), 14.2 (CH_3_). GC‐MS (70 eV, *m/z*): 264, 235, 221, 147, 135, 121, 109, 95, 81, 67, 55.

#### 2.6.8 *15,15‐Diethoxypentadec‐(*E*)‐undec‐2‐enal (* ***10*** *)*

A THF solution (10 mL) of compound **7** (10.0 g, 32.1 mmol) was added dropwise to a suspension of LiAlH_4_ (1.22 g, 32.1 mmol) in THF (20 mL). The resultant mixed solution was subjected to 2 h of stirring under ambient temperature, and then 5 g of Celite and Na_2_SO_4_·10H_2_O mixture (1:1, v/v) was carefully added to quench the reaction, followed by slurry filtering. Next, 20 mL of hexane was used to wash the Celite bed three times. MgSO_4_ was used to dry the integrated organic phase before solvent evaporation at reduced pressure. EMD was used to oxidize the crude product. The dienal **10** was prepared according to a previous description for preparing compound **8** based on compound **7**, producing a 90% yield in two steps (9.02 g, pale‐yellow oil). ^1^H NMR (500 MHz, CDCl_3_, ppm) *δ*: 1.20 (6H, t, *J* = 7.0 Hz, 2CH_3_), 1.26–1.30 (16H, m, 8CH_2_), 1.47–1.53 (2H, m, H‐5), 1.57–1.61 (2H, m, H‐14), 2.30–2.35 (2H, m, H‐4), 3.46–3.50 (2H, m, OCH_2_), 3.61–3.64 (2H, m, OCH_2_), 4.47 (1H, t, *J* = 6.0 Hz, CH), 6.11 (1H, m, H‐2), 6.85 (1H, dt, *J* = 15.5, 6.5 Hz, H‐3), 9.49 (1H, d, *J* = 8.0 Hz, CHO). ^13^C NMR (125 MHz, CDCl_3_, ppm) *δ*: 194.2 (CHO), 159.1 (C‐3), 132.9 (C‐2), 102.9 (CH), 60.8 (CH_2_‐O), 60.8 (CH_2_‐O), 33.6 (C‐15), 32.7 (C‐4), 29.5 (CH_2_), 29.5 (CH_2_), 29.5 (CH_2_), 29.5 (CH_2_), 29.5 (CH_2_), 29.3 (CH_2_), 29.1 (CH_2_), 27.8 (CH_2_), 24.7 (CH_2_), 15.3 (CH_3_), 15.3 (CH_3_).

#### 2.6.9 *(13*E*,15*E*)‐1,1‐Diethoxy‐octadecadienal (* ***11*** *)*

Compound **11** was prepared from compound **10** (3.12 g, 10 mmol) according to a previous description for preparing compound **9** from compound **8**. A mixture of *E*‐**11** and *Z*‐**12** in a 10:1 ratio was obtained from this process, which was then chromatographed (10% silver nitrate SiO_2_; hexane:ethyl acetate, 60:1) to produce the colorless oil **11** (2.03 g, 60% yield). ^1^H NMR (500 MHz, CDCl_3_, ppm) *δ*: 0.99 (3H, t, *J* = 7.5 Hz, CH_3_), 1.20 (6H, t, *J* = 7.5 Hz, 2CH_3_), 1.25–1.28 (16H, m, 8CH_2_), 1.33–1.37 (2H, m, H‐11), 1.58–1.62 (2H, quint, *J* = 7.0 Hz, H‐3), 2.03–2.09 (4H, m, H‐12 and H‐17), 3.46–3.52 (2H, m, OCH_2_), 3.60–3.66 (2H, m, OCH_2_), 4.47 (1H, t, *J* = 6.0 Hz, CH), 5.54–5.63 (2H, m, H‐13 and H‐16), 5.97–6.01 (2H, m, H‐14 and H‐15). ^13^C NMR (125 MHz, CDCl_3_, ppm) *δ*: 133.8 (C‐16), 132.5 (C‐13), 130.3 (C‐14), 129.4 (C‐15), 102.9 (O‐CH‐O), 60.8 (CH_2_‐O), 60.8 (CH_2_‐O), 33.6 (C‐2), 32.6 (C‐12), 29.6 (CH_2_), 29.6 (CH_2_), 29.5 (CH_2_), 29.5 (CH_2_), 29.5 (CH_2_), 29.4 (CH_2_), 29.2 (CH_2_), 29.1 (CH_2_), 25.6 (CH_2_), 24.7 (CH_2_), 15.3 (CH_3_), 15.3 (CH_3_), 13.6 (C‐18).

#### 2.6.10 *(13*E*,15*E*)‐Octadecadienal (* ***3*** *)*

The dienal **3** was prepared from compound **11** (2.03 g, 6.0 mmol) as described before (deprotection of the acetal group by means of oxalic acid dihydrate) at an isomeric purity of >95%, producing 95% yield (1.51 g). ^1^H NMR (500 MHz, CDCl_3_, ppm) *δ*: 0.99 (3H, t, *J* = 7.5 Hz, CH_3_), 1.25–1.29 (16H, m, 8CH_2_), 1.61 (2H, quint, *J* = 7.0 Hz, H‐3), 2.02–2.09 (4H, m, H‐12 and H‐17), 2.41 (2H, td, *J* = 7.5, 1.5 Hz, H‐2), 5.54–5.63 (2H, m, H‐13 and H‐16), 5.97–6.02 (2H, m, H‐14 and H‐15), 9.76 (1H, t, *J* = 2.0 Hz, CHO). ^13^C NMR (125 MHz, CDCl_3_, ppm) *δ*: 202.9 (CHO), 133.8 (C‐16), 132.5 (C‐13), 130.3 (C‐14), 129.4 (C‐15), 43.9 (C‐2), 32.6 (C‐12), 29.5 (CH2), 29.5 (CH_2_), 29.5 (CH_2_), 29.4 (CH_2_), 29.4 (CH_2_), 29.3 (CH_2_), 29.2 (CH_2_), 29.1 (CH_2_), 25.6 (C‐3), 22.1 (C‐17), 13.6 (CH_3_). GC‐MS (70 eV, *m/z*): 264, 235, 221, 207, 147, 135, 121, 109, 95, 81, 67, 55.

#### 2.6.11 *(13*E*,15*Z*)‐1,1‐Diethoxy‐octadecadienal (* ***11*** *′)*

Compound **11′** was prepared from compound **10** (1.56 g, 5 mmol) according to a previous description for preparing compound **9′** based on compound **8**. A mixture of *Z*‐**11′** and *E*‐**11** at 20:1 was obtained during this process, which was chromatographed (10% silver nitrate SiO_2_, hexane:ethyl acetate, 60:1) to produce the colorless oil **11** (1.14 g, 68% yield). ^1^H NMR (500 MHz, CDCl_3_, ppm) *δ*: 0.99 (3H, t, *J* = 7.5 Hz, CH_3_), 1.20 (6H, t, *J* = 7.5 Hz, 2CH_3_), 1.26–1.28 (16H, m, 8CH_2_), 1.37 (2H, m, H‐11), 1.60 (2H, m, H‐3), 2.06 (2H, q, *J* = 8.0 Hz, H‐17), 2.17 (2H, q, *J* = 7.5 Hz, H‐12), 3.45–3.51 (2H, m, OCH_2_), 3.60–3.66 (2H, m, OCH_2_), 4.47 (1H, t, *J* = 6.0 Hz, CH), 5.29 (1H, dt, *J* = 10.5, 7.5 Hz, H‐13), 5.65 (1H, 1H, dt, *J* = 14.5, 7.0 Hz, H‐16), 5.91 (1H, dt, *J* = 14.5, 7.0 Hz, H‐14), 6.27 (1H, ddq, *J* = 14.0, 11.0, 1.5 Hz, H‐15). ^13^C NMR (125 MHz, CDCl_3_, ppm) *δ*: 134.7 (C‐16), 131.6 (C‐13), 128.0 (C‐14), 125.4 (C‐15), 102.9 (O‐CH‐O), 60.8 (CH_2_‐O), 60.8 (CH_2_‐O), 33.6 (C‐2), 32.9 (C‐12), 29.6 (CH_2_), 29.6 (CH_2_), 29.5 (CH_2_), 29.5 (CH_2_), 29.5 (CH_2_), 29.5 (CH_2_), 29.4 (CH_2_), 29.2 (CH_2_), 24.7 (CH_2_), 20.9 (CH_2_), 15.3 (CH_3_), 15.3 (CH_3_), 14.3 (C‐18).

#### 2.6.12 *(13*E*,15*Z*)‐Octadecadienal (* ***4*** *)*

The dienal **4** was prepared from compound **11′** (1.0 g, 2.95 mmol) as previously described (for the deprotection of acetal group by oxalic acid dihydration) at an isomeric purity of >95%, resulting in 96% yield (0.75 g). ^1^H NMR (500 MHz, CDCl_3_, ppm) *δ*: 0.99 (3H, t, *J* = 7.5 Hz, CH_3_), 1.26–1.30 (16H, m, 8CH_2_), 1.61 (2H, quint, *J* = 7.5 Hz, H‐3), 2.08 (2H, q, *J* = 7.5 Hz, H‐17), 2.17 (2H, q, *J* = 7.5 Hz, H‐12), 2.41 (2H, td, *J* = 7.5, 1.5 Hz, H‐2), 5.29 (1H, dt, *J* = 11.0, 7.5 Hz, H‐13), 5.65 (1H, dt, *J* = 14.5, 7.0 Hz, H‐16), 5.91 (1H, t, *J* = 11.0 Hz, H‐14), 6.29 (1H, ddq, *J* = 15.0, 11.0, 1.5 Hz, H‐15), 9.76 (1H, t, *J* = 2.0 Hz, CHO). ^13^C NMR (125 MHz, CDCl_3_, ppm) *δ*: 202.9 (CHO), 134.7 (C‐16), 131.6 (C‐13), 128.0 (C‐14), 125.5 (C‐15), 43.9 (C‐2), 29.5 (CH_2_), 29.5 (CH_2_), 29.5 (CH_2_), 29.5 (CH_2_), 29.4 (CH_2_), 29.4 (CH_2_), 29.3 (CH_2_), 29.2 (CH_2_), 29.1 (CH_2_), 22.1 (C‐3), 20.9 (C‐17), 14.3 (CH_3_).

### 2.7 Field attractiveness test

Field attractiveness tests were conducted in poplar plantations located at Shanghe (Shandong, China) and Suipin (Henan, China) from August to September 2019.

The delta‐shaped traps, each with a sticky board baited with the synthetic compound (1000 μg) at different ratios and 1% butylated hydroxytoluene was injected into a polyvinyl chloride (PVC) capillary tube (inner diameter 0.1 cm, outer diameter 0.18 cm, length 10 cm) (Pherobio Technology Co. Ltd, Beijing, China), were hung from a plastic pole at a height of 3.0 m above the ground at intervals of 30–40 m. The pheromone compounds were dissolved in distilled hexane, then 10 μL of the mixed solution was added to the PVC tubes, with one PVC tube containing distilled hexane solution acting as the reference. In previous tests, no activity was elicited by the antioxidant. The ends of the PVC capillary tubes were sealed through heating. The lures were kept at −20 °C prior to use.

Field experiment 1 was conducted from 26 August to 4 September 2019 in Shanghe, Shandong, China. The lures baited with (13*Z*,15*E*)‐octadecadienal and (13*Z*,15*Z*)‐octadecadienal (ratios 1:0, 1:1, 0:1 and 0:0) were used to determine the most attractive blend of *Z*13,*E*15‐18:Ald and *Z*13,*Z*15‐18:Ald for the male moths. Five duplicates were set for every ratio, a total of 20 traps. All trap catches were measured on a day basis, and each trap in the field test was set according to a randomized block design.

Field experiment 2 was conducted from 1 to 14 September 2019 in Suipin, Henan, China. To illustrate bait effectiveness, all lures were baited with the synthesized compounds at 1:0, 4:1, 3:2, 0:1 and 0:0 ratios. Five duplicates were set for each treatment and the number of moths trapped was determined twice weekly.

### 2.8 Statistical analysis

One‐way analysis of variance was adopted for data analysis, and the means were compared through Tukey's honestly significant difference test (SPSS 17.0, Inc., Chicago, IL, USA). The significance level was *α* = 0.05 for each test.

## 3 RESULTS

### 3.1 Synthesis of the four stereoisomers of 13,15‐octadecadienal

According to our procedure, the acetal derivative, 1,1‐diethoxy‐12‐iododecane **6**, was obtained by three steps (80% yield through 12‐bromo‐1‐decanol). The acetylenic compound **7** was obtained by the alkylation of lithium propargyl alcohol with **6** in HMPT:THF, with a yield of 75% (Scheme 1).

Aldehyde **8** was acquired through oxidizing intermediate **7** with electrolytic manganese dioxide (EMD), resulting in a 93% yield (Scheme 2). *E*‐18,18‐diethoxyoctadec‐3‐en‐5‐yne **9** was generated from a process that started with the Wittig–Schlosser reaction (Scheme 2). Aldehyde **8** was converted to (*E*)‐18,18‐diethoxyoctadec‐3‐en‐5‐yne **9** (*E*‐**9**/*Z*‐**9′** = 7:1) with *n*‐propyl triphenylphosphonium bromide using *n*‐BuLi as the base. *Z*‐18,18‐diethoxyoctadec‐3‐en‐5‐yne **9′** (*Z*‐**9′**/*E*‐**9** = 16:1) was prepared by the normal Wittig reaction. Aldehyde **8** was subjected to a (*Z*)‐selective Wittig reaction using an ylide prepared from pentyltriphenylphosphonium bromide, with potassium bis(trimethylsilyl)amide used as the base (Scheme 2). **9** and **9'**were subjected to (*Z*)‐selective reduction of the triple bond separately by the hydroboration‐protonolysis process using dicyclohexylborane in hexane. The acetal group was removed under acidic conditions (aqueous oxalic acid). After purification by column chromatography using silica gel impregnated with 10% silver nitrate, the (*Z*,*E*)‐diene **1** (63% based on **9**, isomeric purity >95%) and (*Z*,*Z*)‐diene **2** (62% based on **9′**, isomeric purity >96%) were obtained.

The normal (*E*,*Z*‐**11′**/*E*,*E*‐**11** = 20:1, 68% yield) and Schlosser–Wittig conditions (*E*,*E*‐**11**/*E*,*Z*‐**11′** = 10:1, 66% yield) were adopted for the separate formation of the protected *E*,*E*‐**11** and *E*,*Z*‐**11′** dienals (Scheme 3). The isomeric purity levels of *E*,*E*‐**3** and *E*,*Z*‐**4** increased to >95% following deprotection and purification.

Finally, *Z*13,*E*15‐18:Ald, *Z*13,*Z*15‐18:Ald, *E*13,*E*15‐18:Ald, and *E*13,*Z*15‐18:Ald were successfully synthesized in eight steps.

### 3.2 Chromatographic analysis

**Figure 1:**
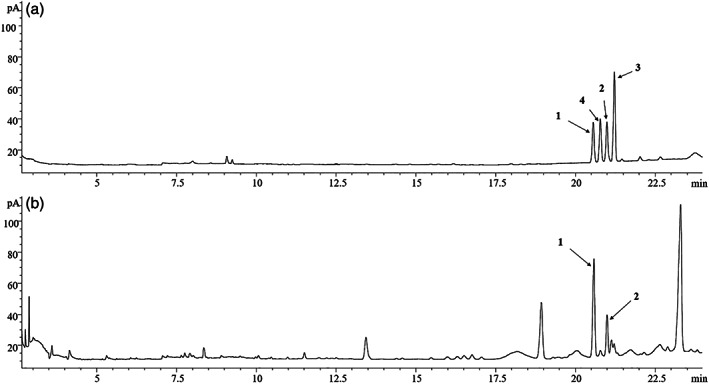
(a) GC analysis of a mixture of synthetic compounds. (b) GC analysis of the pheromone extracts of female *M. siversi*. **1**, (13*Z*,15*E*)‐octadecadienal; **2**, (13*Z*,15*Z*)‐octadecadienal; **3**, (13*E*,15*E*)‐octadecadienal; **4**, (13*E*,15*Z*)‐octadecadienal.

The GC spectra of pheromone extracts were compared with those of the synthesized compounds (*Z*13,*E*15‐18:Ald **1**: Retention time 20.55 min; *Z*13,*Z*15‐18:Ald **2**: Retention time 20.98 min; *E*13,*E*15‐18:Ald **3**: Retention time 21.22 min; *E*13,*Z*15‐18:Ald **4**: Retention time 20.77 min) (Fig. 1(a),(b)), It was found that *Z*13,*E*15‐18:Ald **1** was the major component (~14.72 ± 8.68 ng/gland) and existed together with a very small amount of *Z*13,*Z*15‐18:Ald **2** at a ratio of about 7:3.

**Figure 2:**
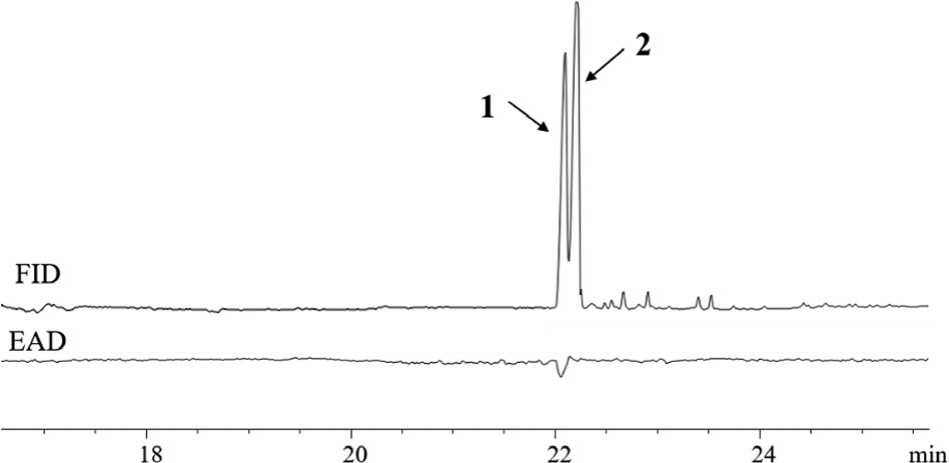
Response of *M. siversi* male antennae to synthetic standards detected byGC‐EAD) **1**, (13*Z*,15*E*)‐octadecadienal; **2**, (13*Z*,15*Z*)‐octadecadienal.

The male antennal response to a mixture of *Z*13,*E*15‐18:Ald **1** and *Z*13,*Z*15‐18:Ald **2** was tested (Fig. 2). As suggested by the GC‐EAD results, *Z*13,*E*15‐18:Ald **1** was the only electrophysiologically active compound (Retention time 22.02 min), whereas *Z*13,*Z*15‐18:Ald **2** elicited no activity (22.33 min) (Fig. 2).

### 3.3 Field attractiveness test

**Figure 3:**
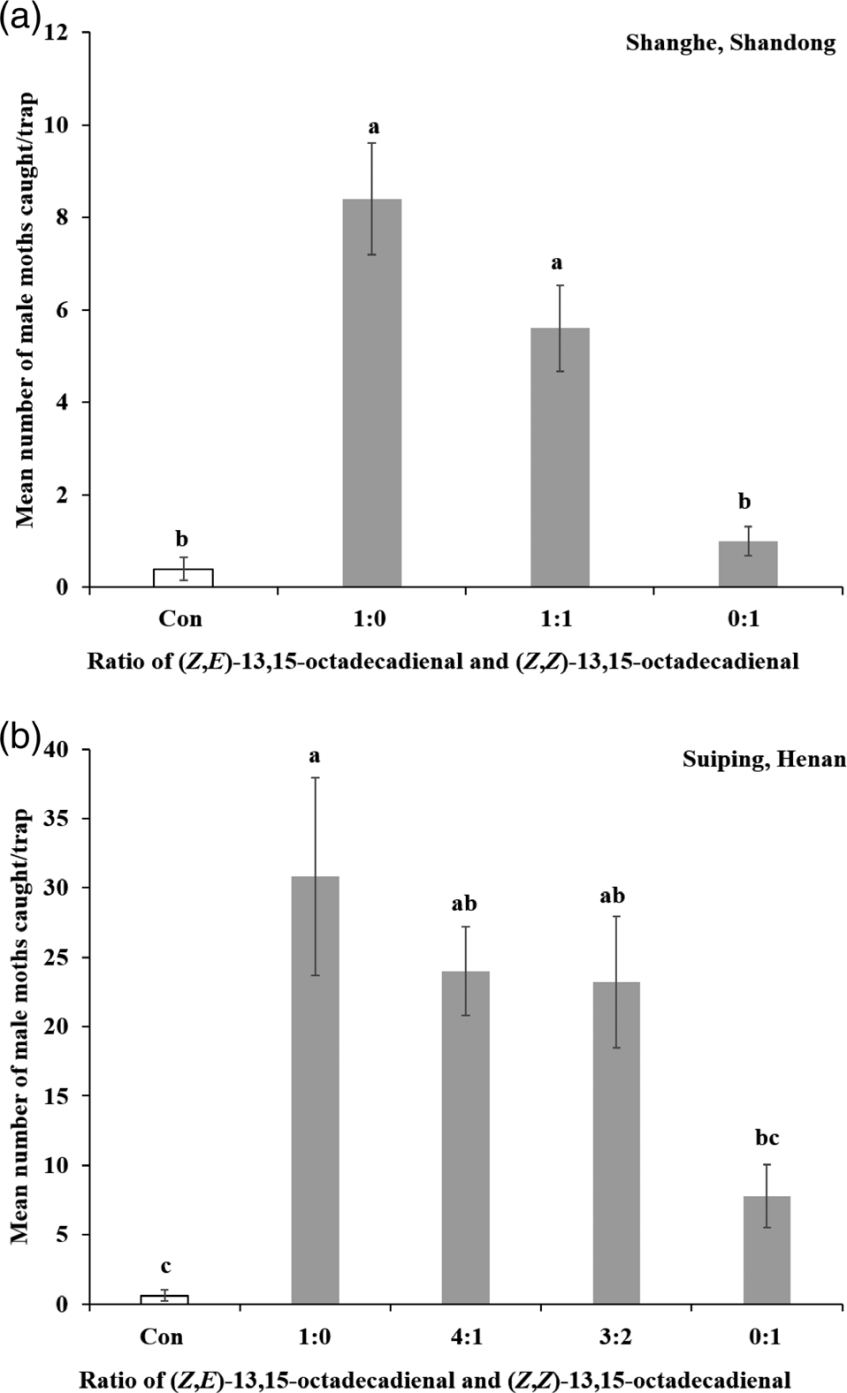
The number of *M. siversi* male moths trapped (mean ± SE) in lures containing the synthesized compounds (dose = 1000 μg). (a) In Shanghe, Shandong (116.57°34.14′ E, 37.20°17.38′ N) from 26 August to 4 September 2019. Five duplicates were set for every treatment (*F*_(3,16)_ = 23.543, *P* < 0.001). (b) In Suipin, Henan (113.23°40.18′ E, 33.15°26.23′ N) from 1 to 14 September 2019. Five duplicates were set for every treatment (*F*_(4,20)_ = 8.866, *P* < 0.001). Columns designated with different lowercase letters indicate significant differences (*P* < 0.05).

In field experiment 1, the attractiveness of *Z*13,*E*15‐18:Ald and *Z*13,*Z*15‐18:Ald was tested and showed significant differences among all treatments (*F*_(3,16)_ = 23.543, *P* < 0.001) (Fig. 3(a)). Traps baited with *Z*13,*Z*15‐18:Ald alone caught a small number of males and there was no difference compared with the control. However, the number of catches increased significantly when *Z*13,*E*15‐18:Ald was added. Traps baited with *Z*13,*E*15‐18:Ald alone caught more males than those baited with*Z*13,*Z*15‐18:Ald alone and the binary blend (1:1). The results show that the presence of *Z*13,*E*15‐18:Ald significantly affects the attractiveness of the traps.

In field experiment 2, to further verify the attractiveness of *Z*13,*E*15‐18:Ald, and the binary blends of *Z*13,*E*15‐18:Ald and *Z*13,*Z*15‐18:Ald were tested in another field experiment (*F*_(4,20)_ = 8.866, *P* < 0.001) (Fig. 3(b)). The traps baited with *Z*13,*Z*15‐18:Ald alone caught fewer males among the tested lures at different ratios. Meanwhile, the numbers of catches of the binary blends were almost the same as those in traps baited with *Z*13,*E*15‐18:Ald. The traps baited with *Z*13,*E*15‐18:Ald alone caught the most males among the tested lures. These results indicate that *Z*13,*E*15‐18:Ald is the key sex pheromone component of *M. siversi*. Although *Z*13,*Z*15‐18:Ald exists in the pheromone extracts of *M. siversi*, it might make no difference to the attractiveness of *Z*13,*E*15‐18:Ald.

## 4 DISCUSSION

In Lepidoptera, sex pheromone components play an important role in chemical communication and reproductive behavior. There are considerable differences in the chemical structures of the species‐specific pheromones. Typically, the varied pheromones are mainly categorized by their chemical structures.^19^, ^20^ Type I pheromone components of 10 species from Notodontid have been described, most of which contain C16 (*Z*,*Z*)‐dienes or conjugated enyne moieties.^18^ In *Thaumetopoea bonjeani*, the sex pheromone represents a mixture composed of *Z*11,*Z*13‐16:Ald and *Z*11,*Z*13‐16:OH.^21^ For *T. pityocampa*, *T. wilkinsoni*, and *T. processionea*, *Z*13,Y11‐16:Ac represents the critical sex pheromone.^22^, ^23^, ^24^ Similarly, *Z*13,Y11‐16:Ald is the critical sex pheromone in *Heterocampa guttivitta*,^25^ while *Z*11,*Z*13‐16:Ald and *Z*11,*Z*13‐16:Ac are the critical sex pheromones in *Notodonta dromedaries* and *N. torva*, respectively.^26^, ^27^ Interestingly, there were no traces of C16‐dienes or conjugated enynes in the pheromone gland extracts of *M. siversi*, where 13,15‐octadecadienal was the only active component detected. Therefore, *M. siversi* appears to be the only species of Notodontidae that uses octadecadienal as the sex pheromone component. In this study, *Z*13,*E*15‐18:Ald, *Z*13,*Z*15‐18:Ald, *E*13,*E*15‐18:Ald, and *E*13,*Z*15‐18:Ald were synthesized by geometrically selective approaches. Based on the electrophysiological and field experimental results, *Z*13,*E*15‐18:Ald was found to be an attractant component in the blend of sex pheromones of *M. siversi*.

The unsaturated aliphatic aldehyde 13,15‐octadecadienal consists of a terminal formyl group and a 1,3‐diene system, which act as the critical functional groups for the recognition of the sex pheromone components of *M. siversi*. As far as we know, there are few reports on the unsaturated dienyl type I pheromone at C‐13 and C‐15 in Lepidoptera. Typically, only *Z*13,*Z*15‐18:Ald is suggested to be a component of the sex pheromone in *Thaumetopoea solitaria*.^28^

The formyl group was first introduced as an acetal derivative at the early synthesis period, and this avoided isomerizing the adjoint diene system from oxidation.^29^ According to our procedure, the acetal derivative 1,1‐diethoxy‐12‐iododecane was obtained in three steps.

The presence of the 1,3‐diene system is quite common in sex pheromones of Lepidoptera. For instance, the derivatives of (5*Z*,7*E*)‐ and (5*E*,7*Z*)‐dodecadienol are the active sex pheromone components of pine caterpillars.^30^, ^31^, ^32^, ^33^ In addition, (11*Z*,13*Z*)‐hexadecadienal, together with related derivatives, are the critical sex pheromones and attractants in Notodonfidae.^18^, ^34^ (8*E*,10*E*)‐dodecadienyl acetate synergizes the codlemone attraction to male *Cydia pomonella*, and it is also the sex pheromone of *Cydia toreuta* (Grote) and *Melissopus latiferreanus*.^35^, ^36^, ^37^

Wittig olefination is an effective protocol to construct the 1,3‐diene system of sex pheromone components.^38^, ^39^, ^40^, ^41^ In the normal Wittig reaction, the unstabilized ylides are produced primarily through those *erythro* betaine intermediates, resulting in the production of *Z*‐alkene products.^42^, ^43^, ^44^ By contrast, the *E*‐alkene products are mainly produced by the Wittig–Schlosser reaction via the *threo* betaine intermediates.^45^, ^46^, ^47^ Accordingly, in this study, the *E* configuration of the enyne and diene were constructed by the Wittig–Schlosser condition,^45^ whereas the *Z* configurations were prepared through the normal Wittig reaction.^48^, ^49^

Although *Z*13,*Z*15‐18:Ald was presented in the pheromone extracts of female *M. siversi*, it elicited no electrophysiological activity. In the field experiments, traps baited with the *Z*,*Z* isomer alone caught a few males, which might result from the presence of 3.6% of *Z*,*E* isomer in the bait. On the whole, the *Z*,*Z* isomer appeared to have no influence on the number of catches when it was mixed with the *Z*,*E* isomer at various ratios. More research is warranted for verification of these results.

## 5 CONCLUSION

Our results indicate that *Z*13,*E*15‐18:Ald, a heretofore undescribed natural product, is the most active sex pheromone component of *M. siversi*. Traps baited with this synthesized sex pheromone component can be used in *M. siversi* monitoring or even in mating disruption. Further work will focus on the development of more convenient and efficient traps.

## Supporting information


**Appendix S1**: Supporting information
