# Supplementary material for: Synthesis and bioactivity of (13Z,15E)‐octadecadienal: A sex pheromone component from Micromelalopha siversi Staudinger (Lepidoptera: Notodontidae)
Source: Pest Manag Sci. 2020 Aug 31;77(1):264–72. doi: 10.1002/ps.6015 (PMC7754109; doi:10.1002/ps.6015)
Supplement: Supplementary file 1 — Appendix S1: Supporting information [file PS-77-264-s001.doc]

**Supplementary information**

**Synthesis and bioactivity of (13*Z*,15*E*)-octadecadienal: a sex pheromone component from *Micromelalopha siversi* Staudinger (Lepidoptera: Notodontidae)**

Fu Liu^1^, Li Guo^1^, Su-Fang Zhang^1^, Xiang-Bo Kong^1^, and Zhen Zhang^1^, *

^1^ Research Institute of Forest Ecology, Environment and Protection, Chinese Academy of Forestry, Key Laboratory of Forest Protection of National Forestry and Grassland Administration, Beijing 100091, China

^2^ Academy of Biological Science and Engineering, Xingtai University, Xingtai 054001, China

**NMR data for the synthetic compounds**

For compound **6**

S1-1 The ^1^H NMR spectrum of compound **6**

S1-2 The ^13^C NMR spectrum of compound **6**

For compound **7**

S1-3 The ^1^H NMR spectrum of compound **7**

S1-4 The ^13^C NMR spectrum of compound **7**

For compound **8**

S1-5 The ^1^H NMR spectrum of compound **8**

S1-6 The ^13^C NMR spectrum of compound **8**

For compound **9**

S1-7 The ^1^H NMR spectrum of compound **9**

S1-8 The ^13^C NMR spectrum of compound **9**

For compound **1**

S1-9 The ^1^H NMR spectrum of compound **1**

S1-10 The ^13^C NMR spectrum of compound **1**

For compound **9′**

S1-11 The ^1^H NMR spectrum of compound **9′**

S1-12 The ^13^C NMR spectrum of compound **9′**

For compound **2**

S1-13 The ^1^H NMR spectrum of compound **2**

S1-14 The ^13^C NMR spectrum of compound **2**

For compound **10**

S1-15 The ^1^H NMR spectrum of compound **10**

S1-16 The ^13^C NMR spectrum of compound **10**

For compound **11**

S1-17 The ^1^H NMR spectrum of compound **11**

S1-18 The ^13^C NMR spectrum of compound **11**

For compound **3**

S1-19 The ^1^H NMR spectrum of compound **3**

S1-20 The ^13^C NMR spectrum of compound **3**

For compound **11′**

S1-21 The ^1^H NMR spectrum of compound **11′**

S1-22 The ^13^C NMR spectrum of compound **11′**

For compound **4**

S1-23 The ^1^H NMR spectrum of compound **4**

S1-24 The ^13^C NMR spectrum of compound **4**

**EI Mass spectra of the four isomers of 13,15-octadecadienal**

S1-25 EI Mass spectra of compound **1**

S1-26 EI Mass spectra of compound **2**

S1-27 EI Mass spectra of compound **3**

S1-28 EI Mass spectra of compound **4**


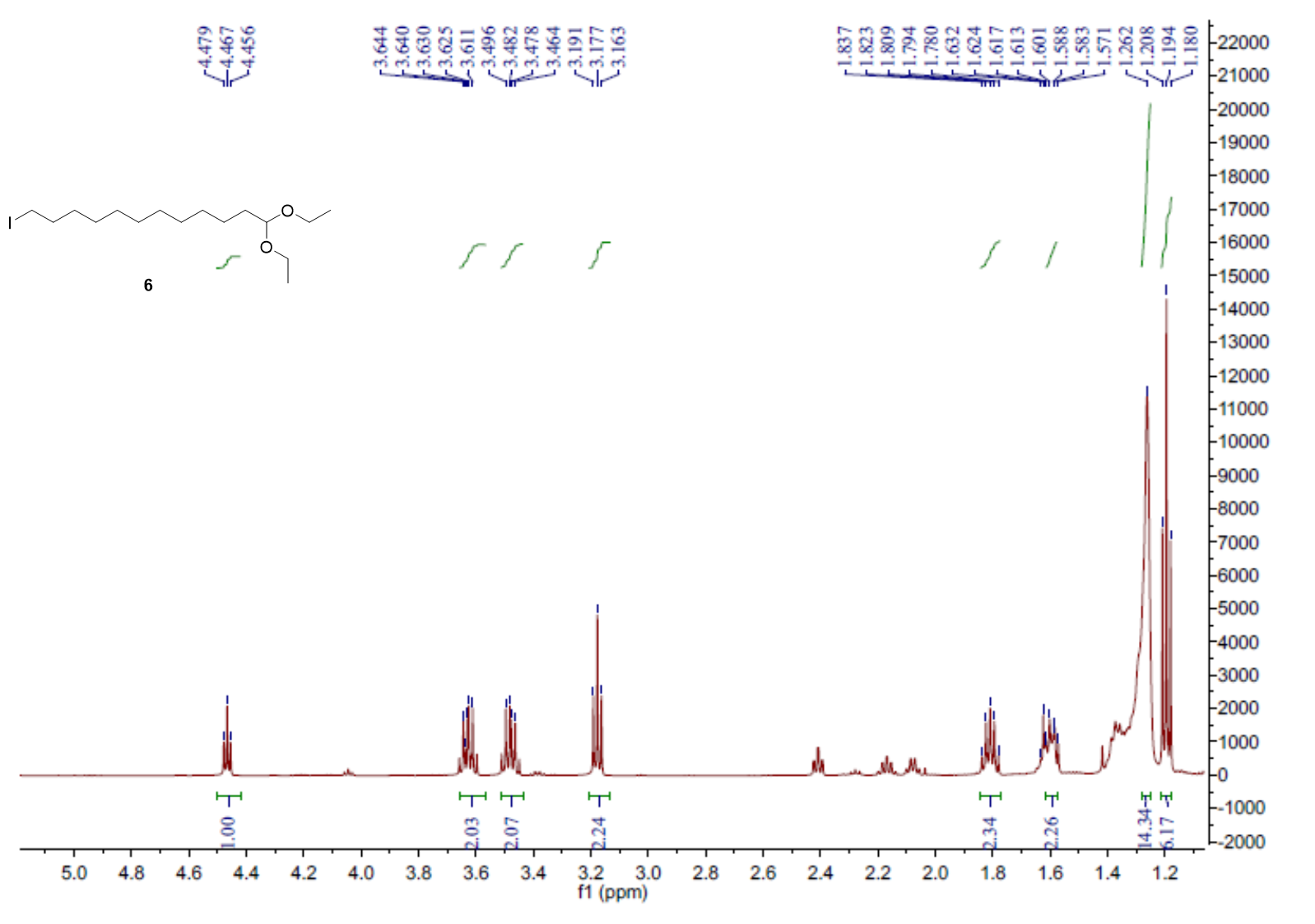


S1-1 The ^1^H NMR spectrum of compound **6**


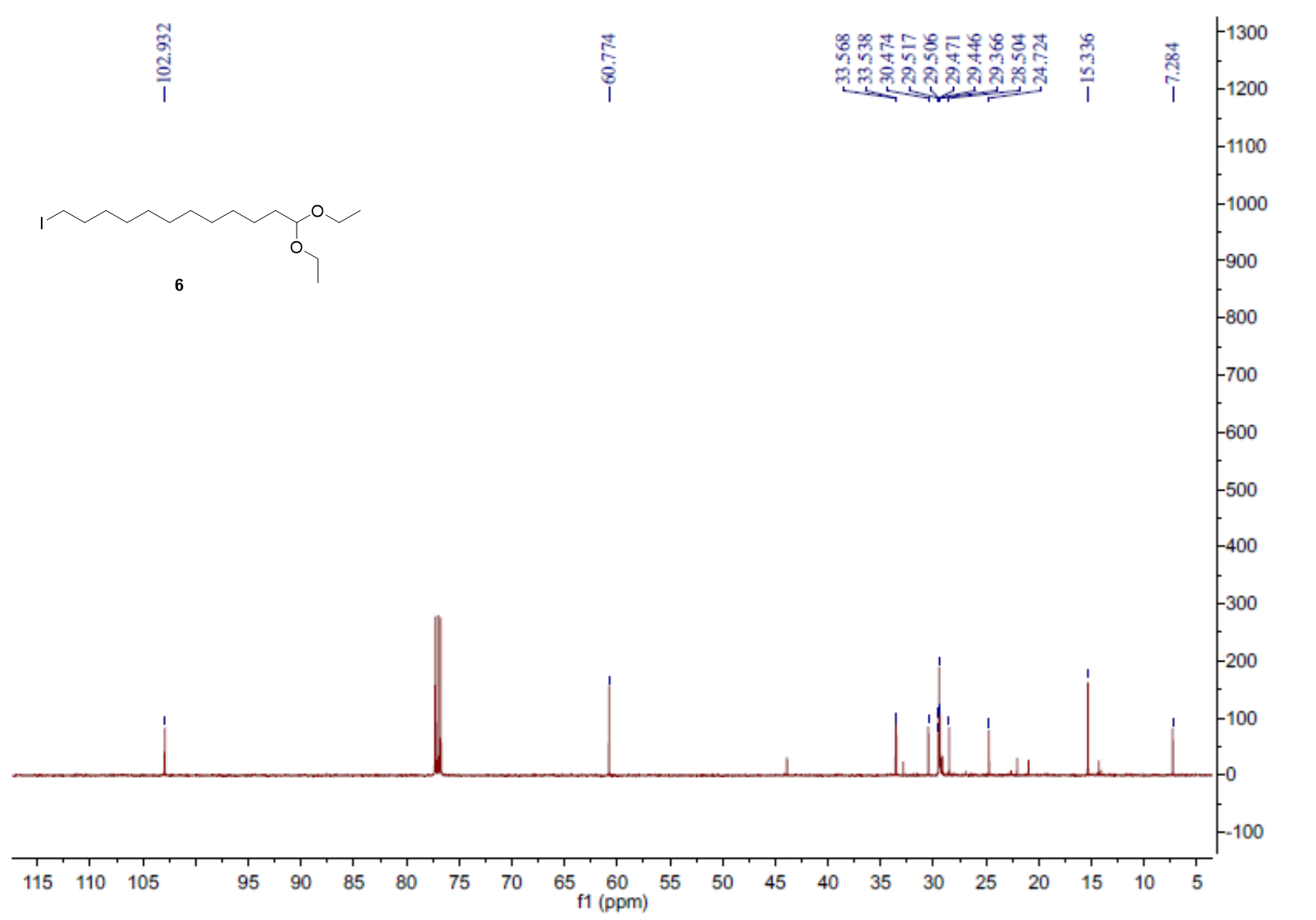


S1-2 The ^13^C NMR spectrum of compound **6**


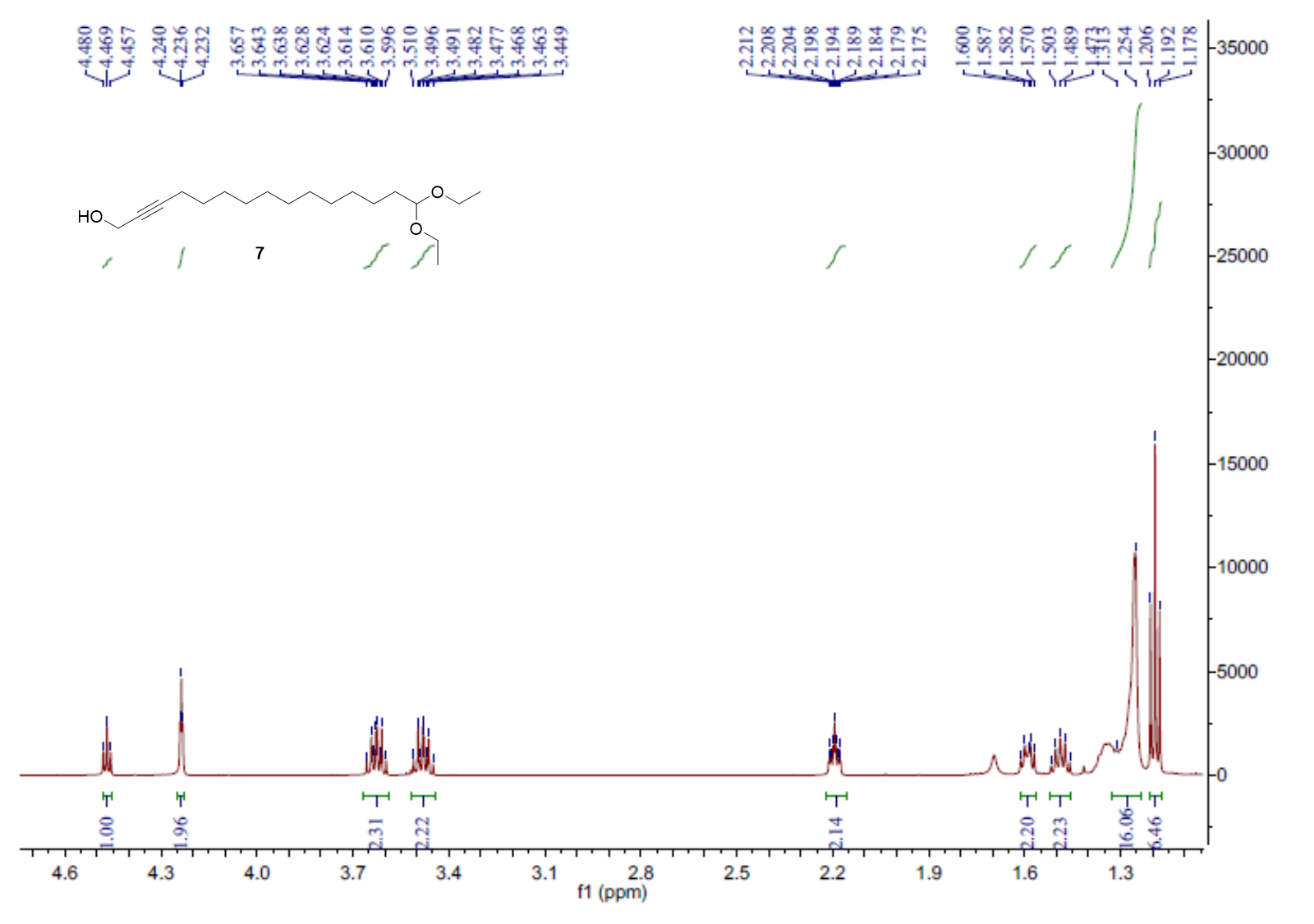


S1-3 The ^1^H NMR spectrum of compound **7**


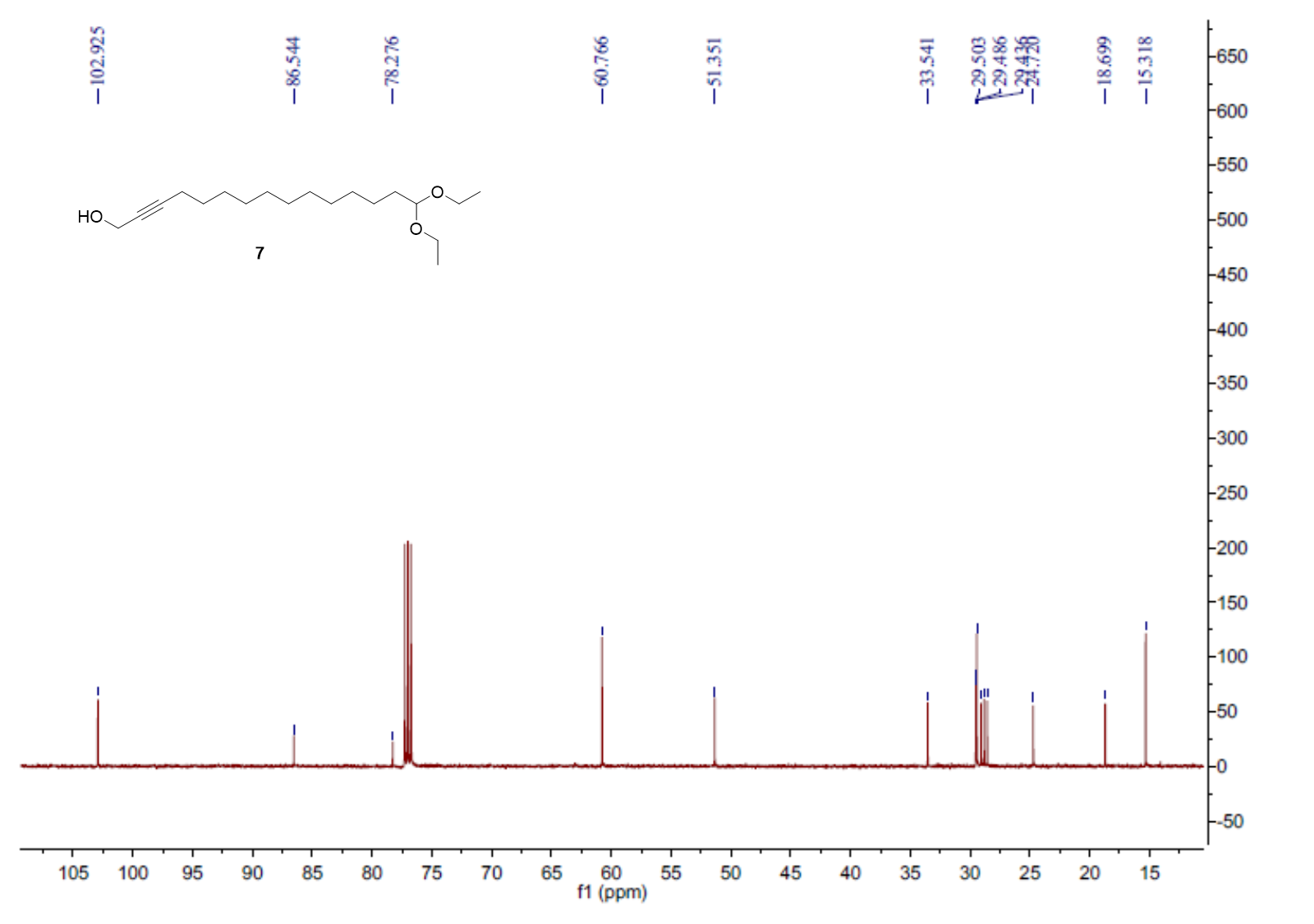


S1-4 The ^13^C NMR spectrum of compound **7**


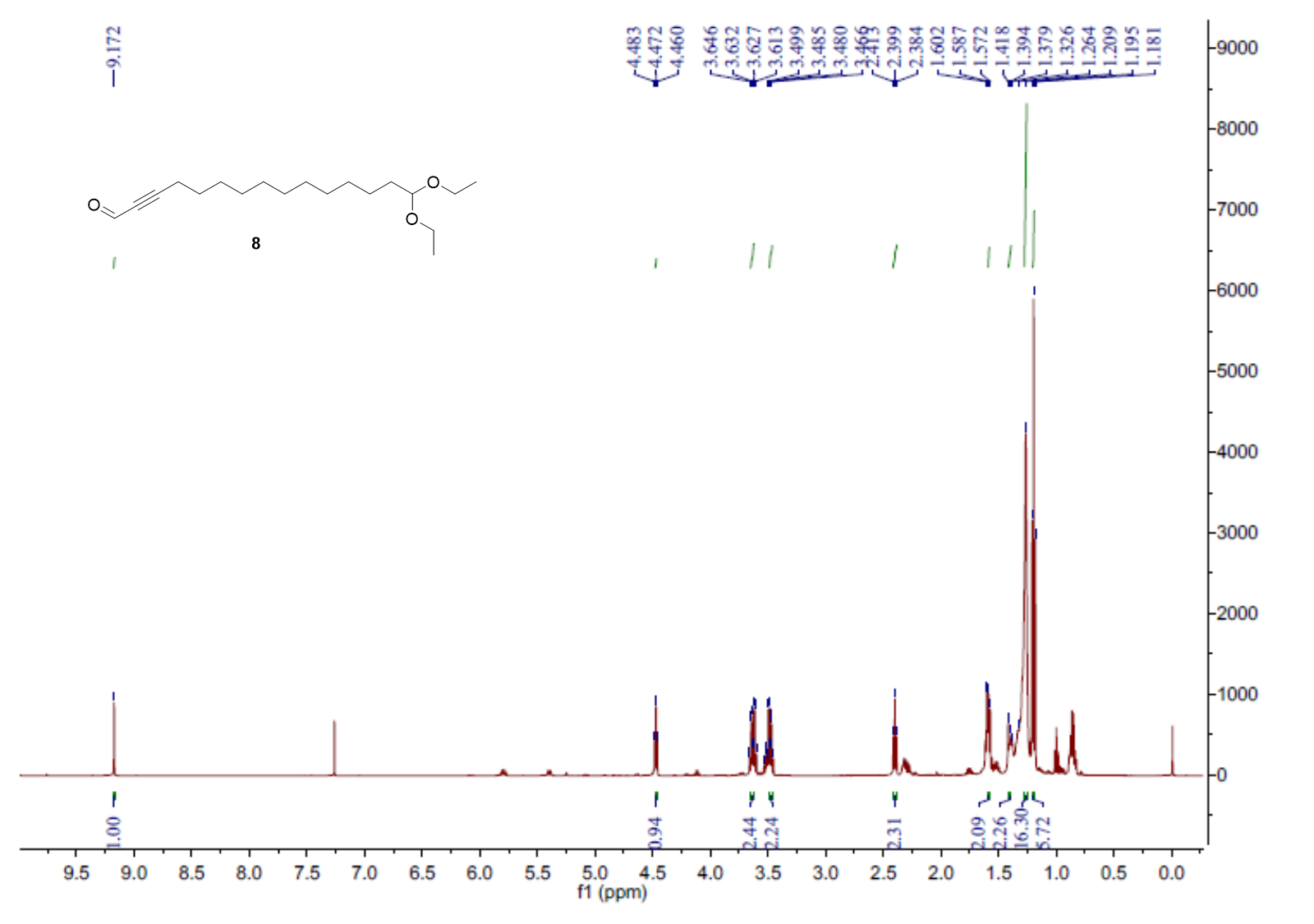


S1-5 The ^1^H NMR spectrum of compound **8**


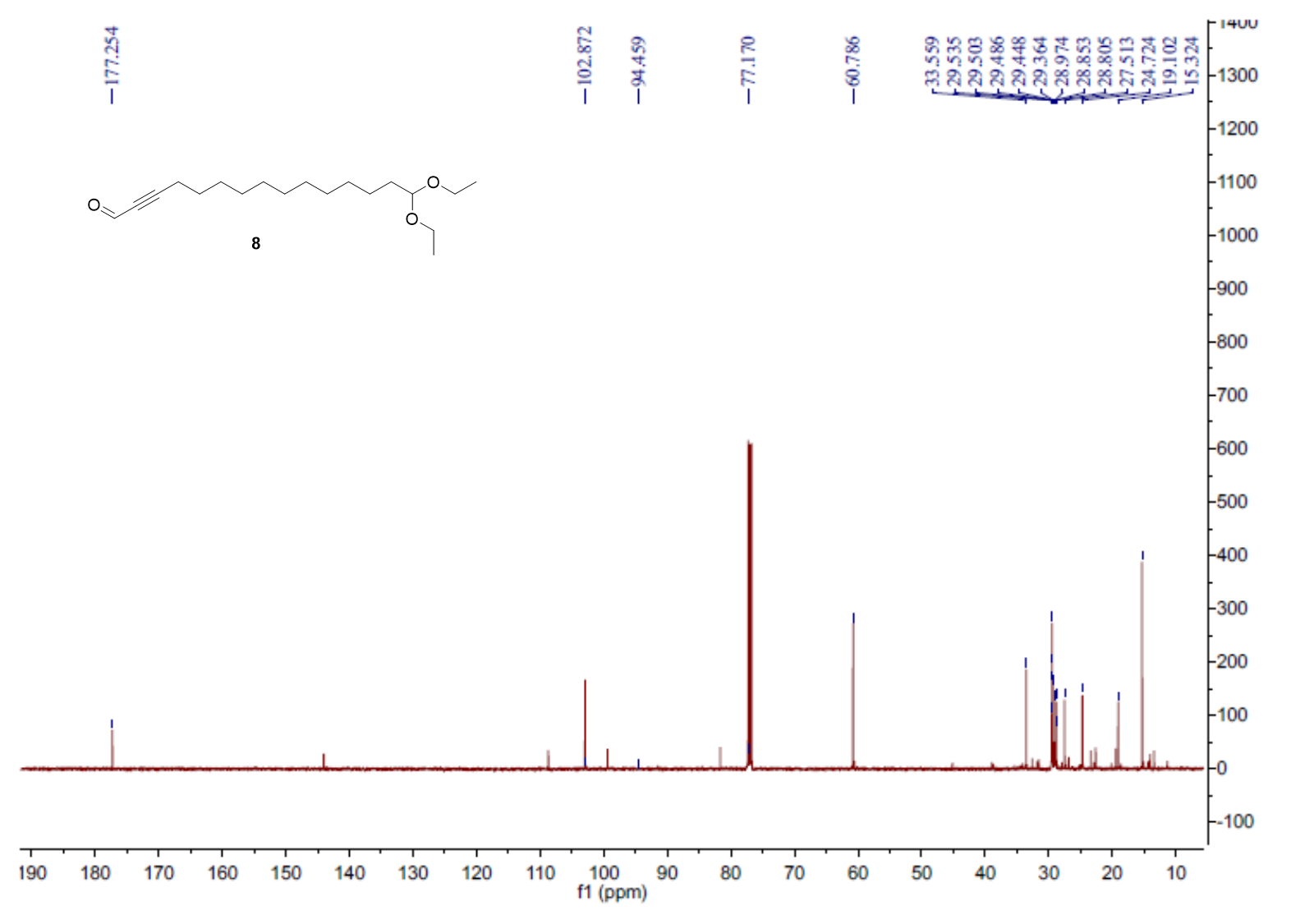


S1-6 The ^13^C NMR spectrum of compound **8**


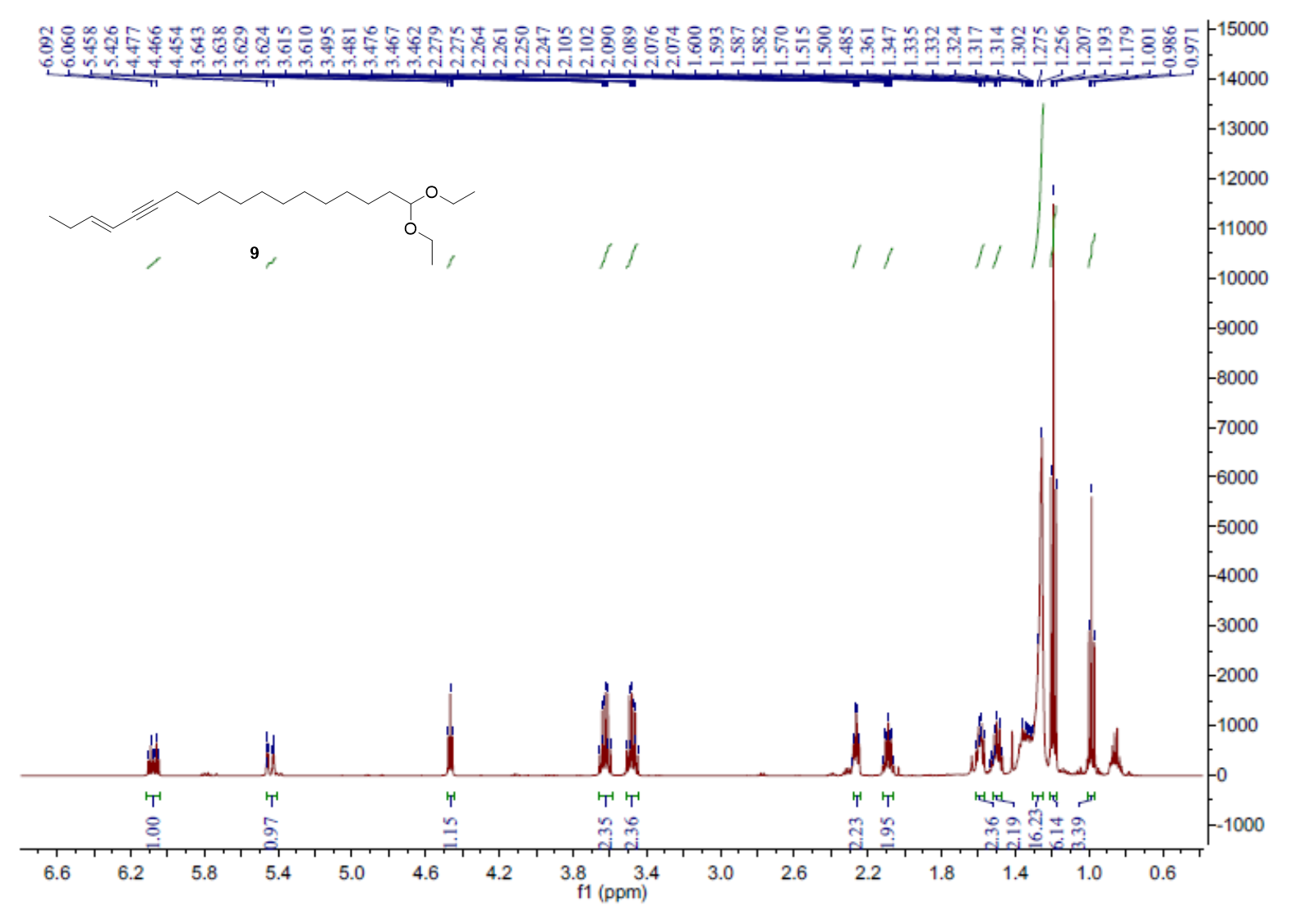


S1-7 The ^1^H NMR spectrum of compound **9**


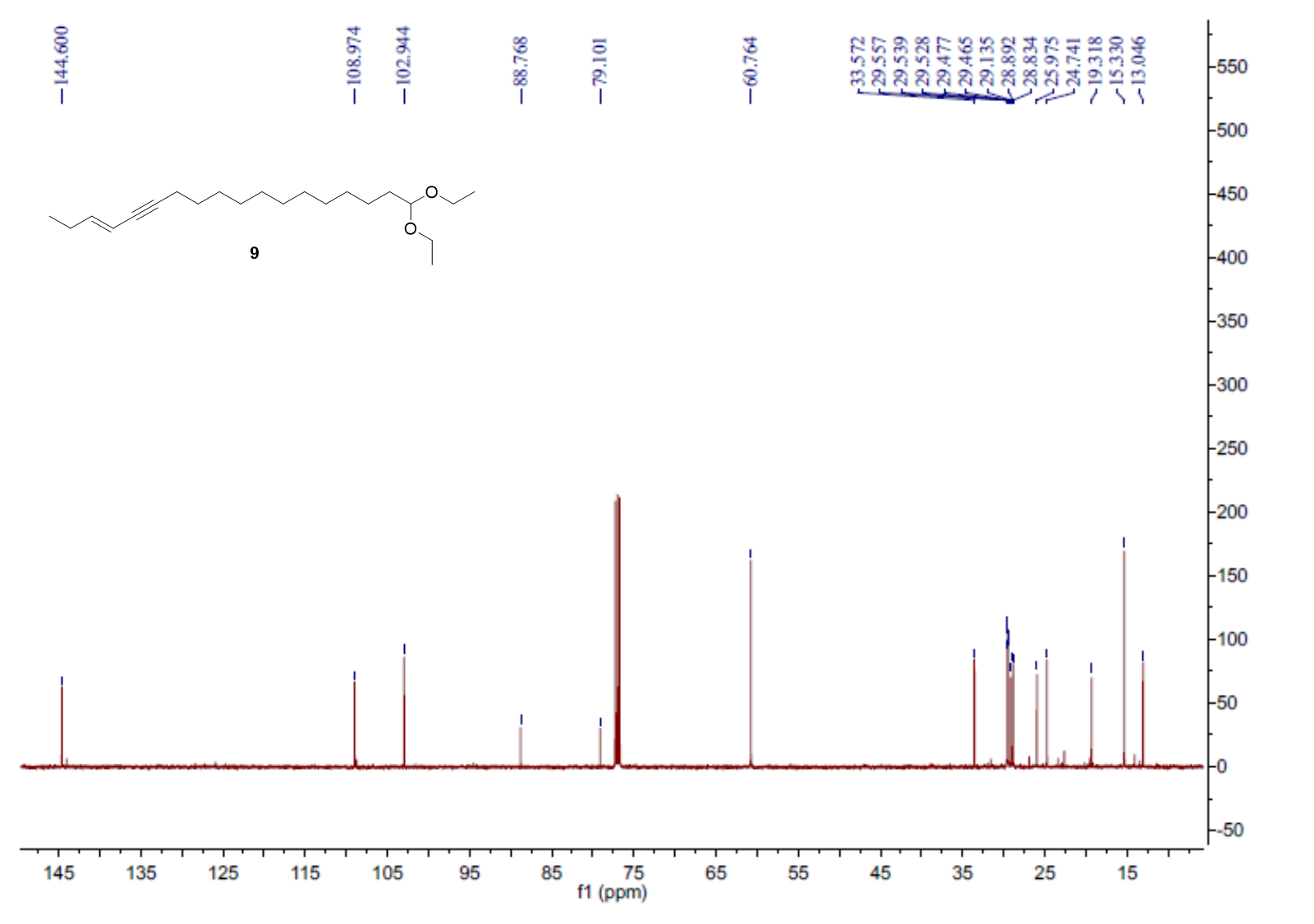


S1-8 The ^13^C NMR spectrum of compound **9**


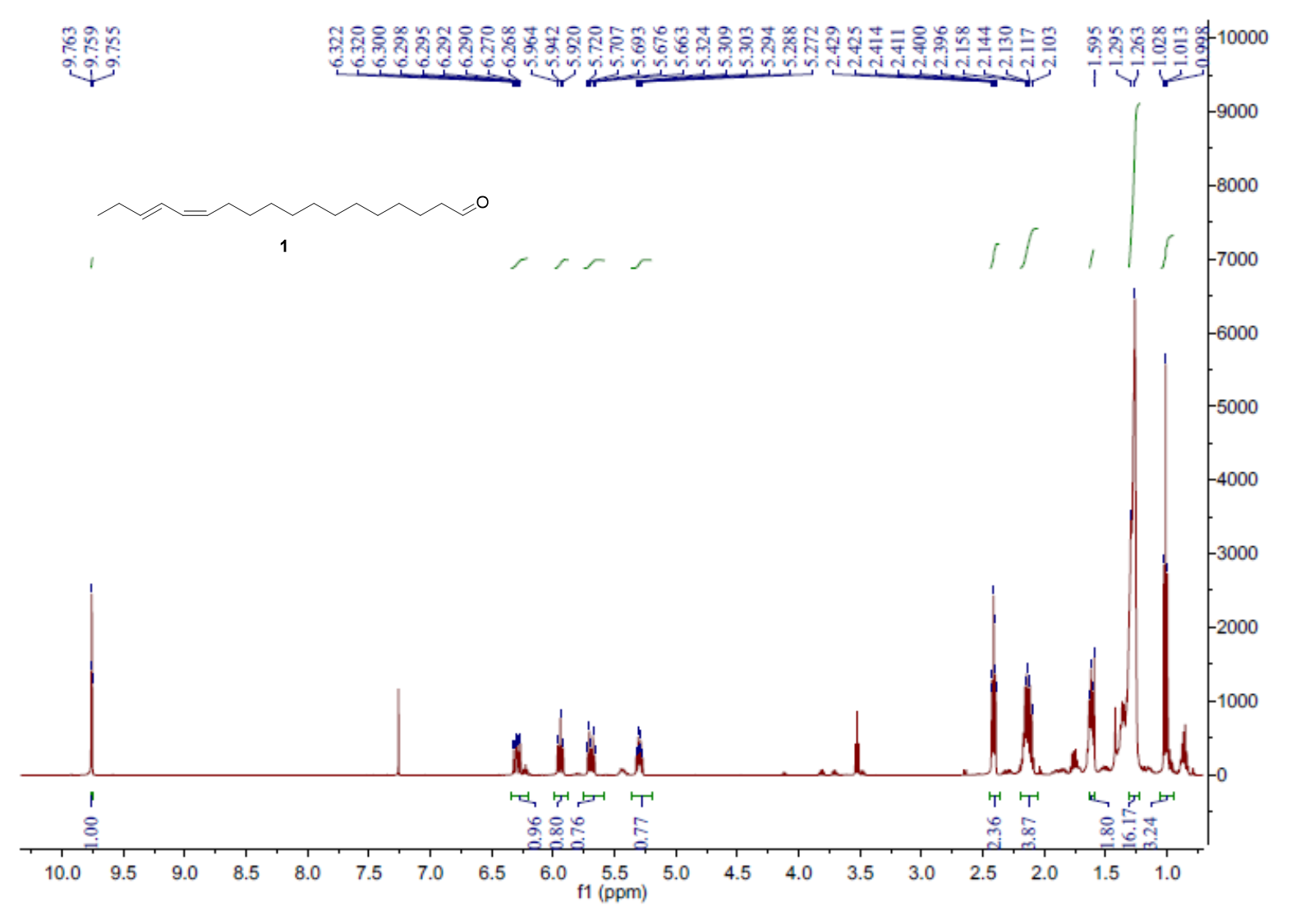


S1-9 The ^1^H NMR spectrum of compound **1**


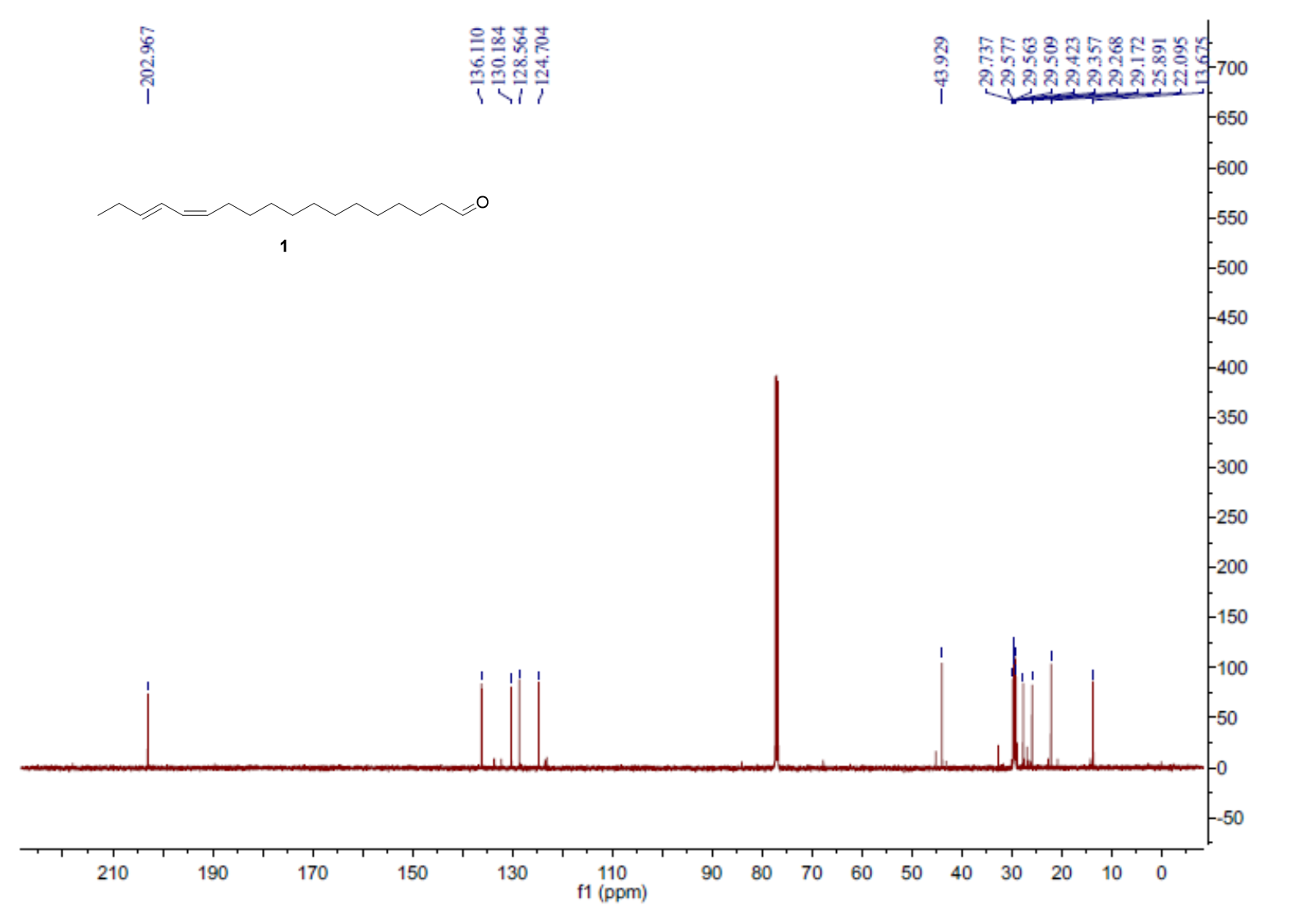


S1-10 The ^13^C NMR spectrum of compound **1**


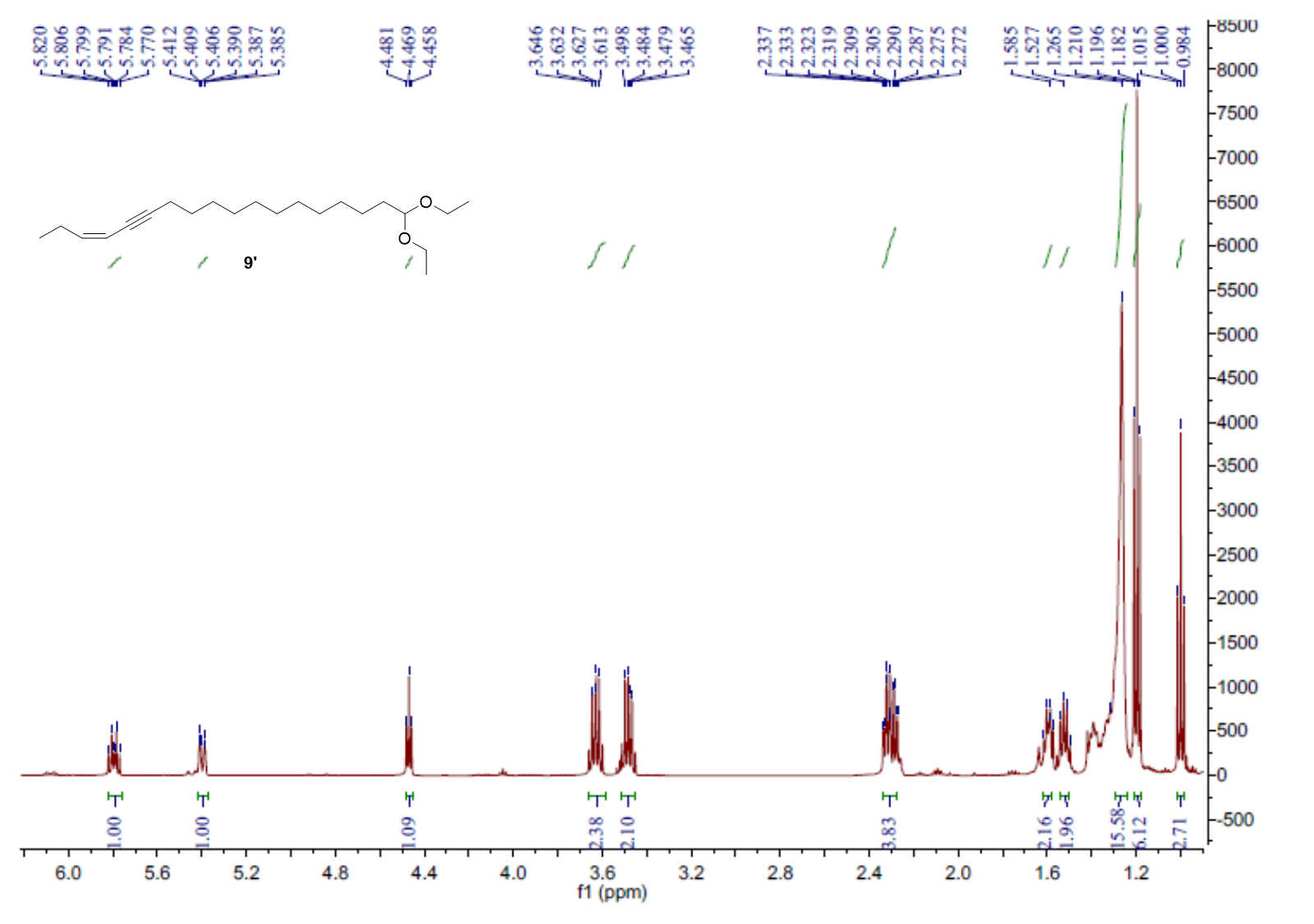


S1-11 The ^1^H NMR spectrum of compound **9′**


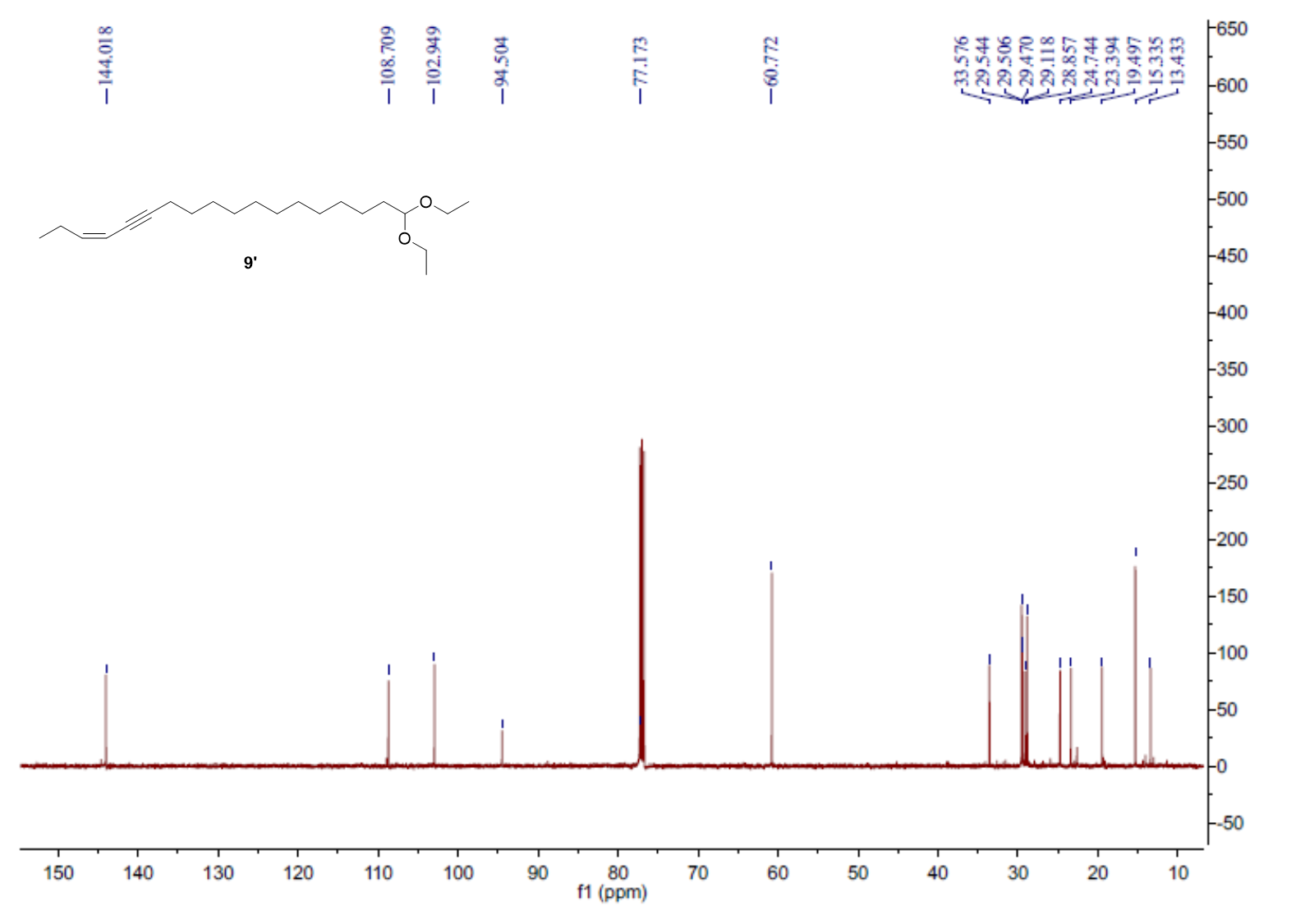


S1-12 The ^13^C NMR spectrum of compound **9′**


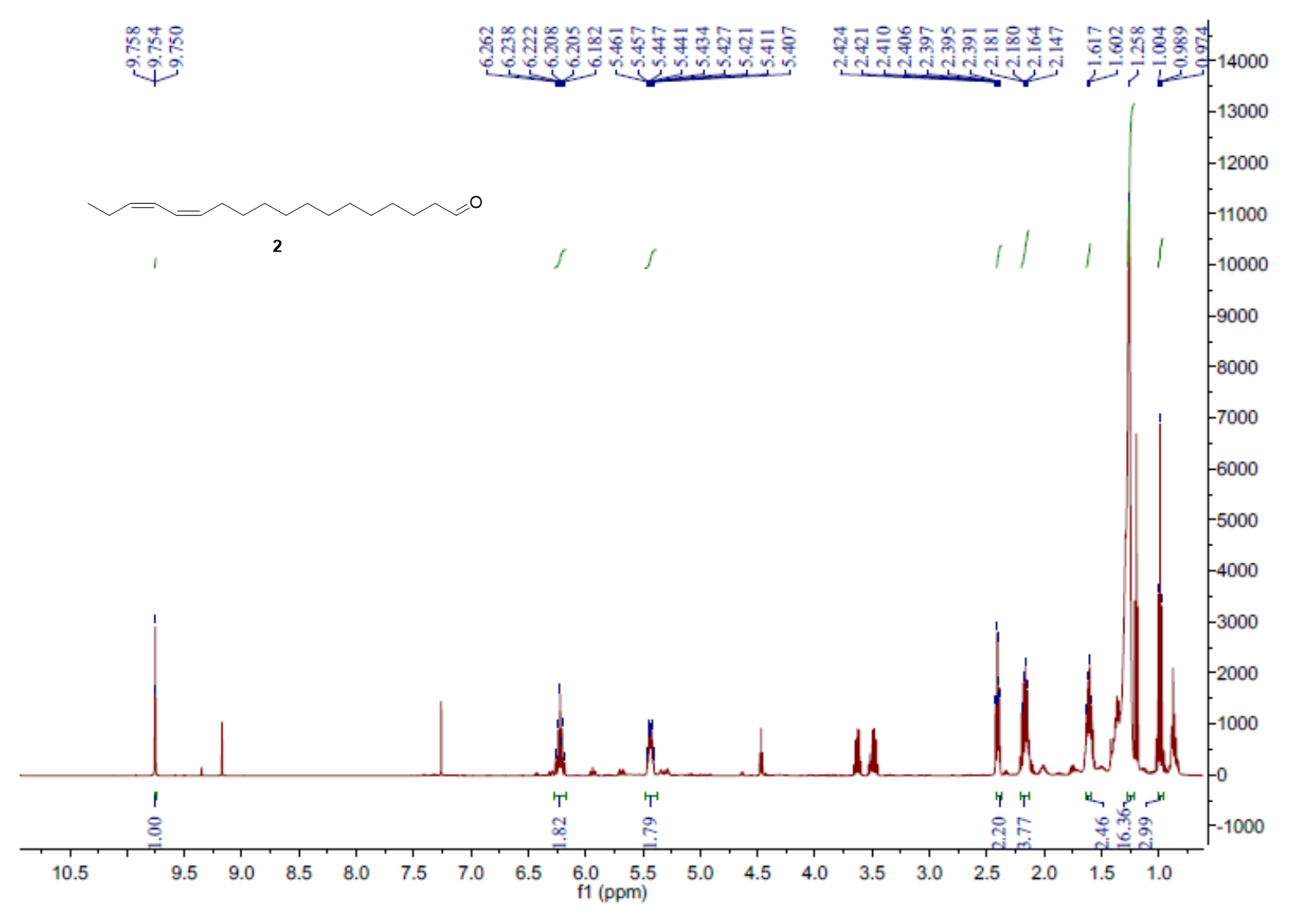


S1-13 The ^1^H NMR spectrum of compound **2**


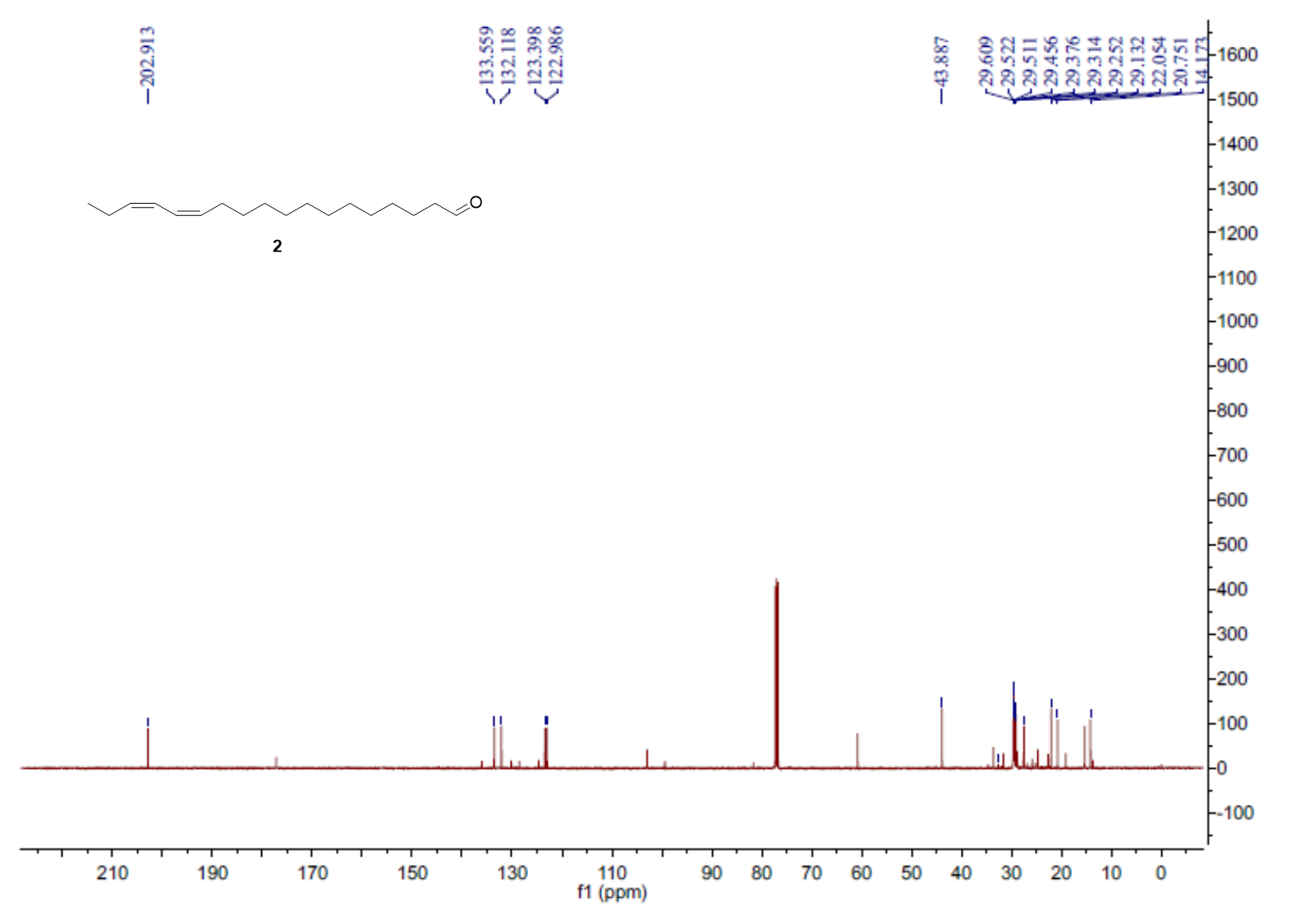


S1-14 The ^13^C NMR spectrum of compound **2**


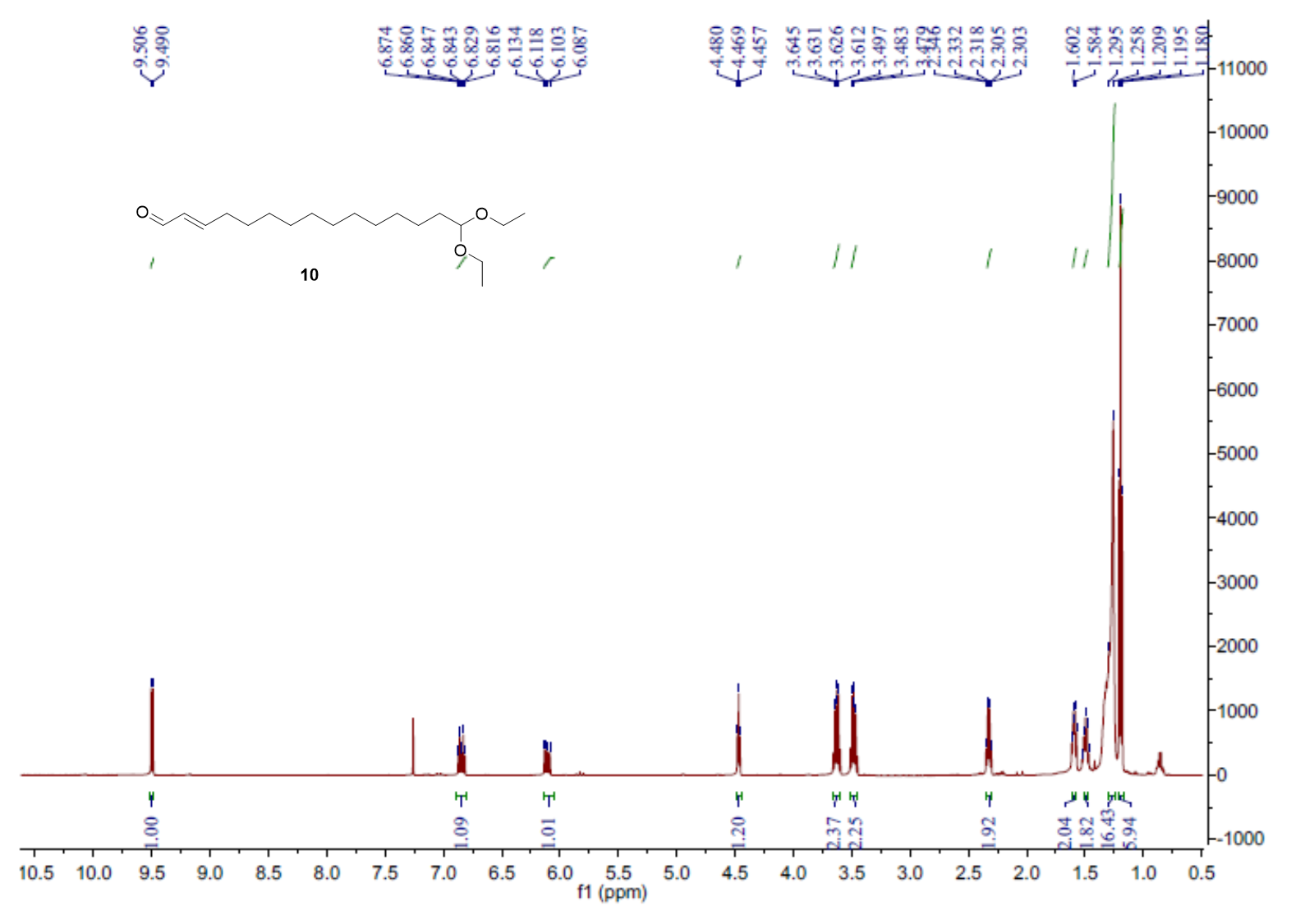


S1-15 The ^1^H NMR spectrum of compound **10**


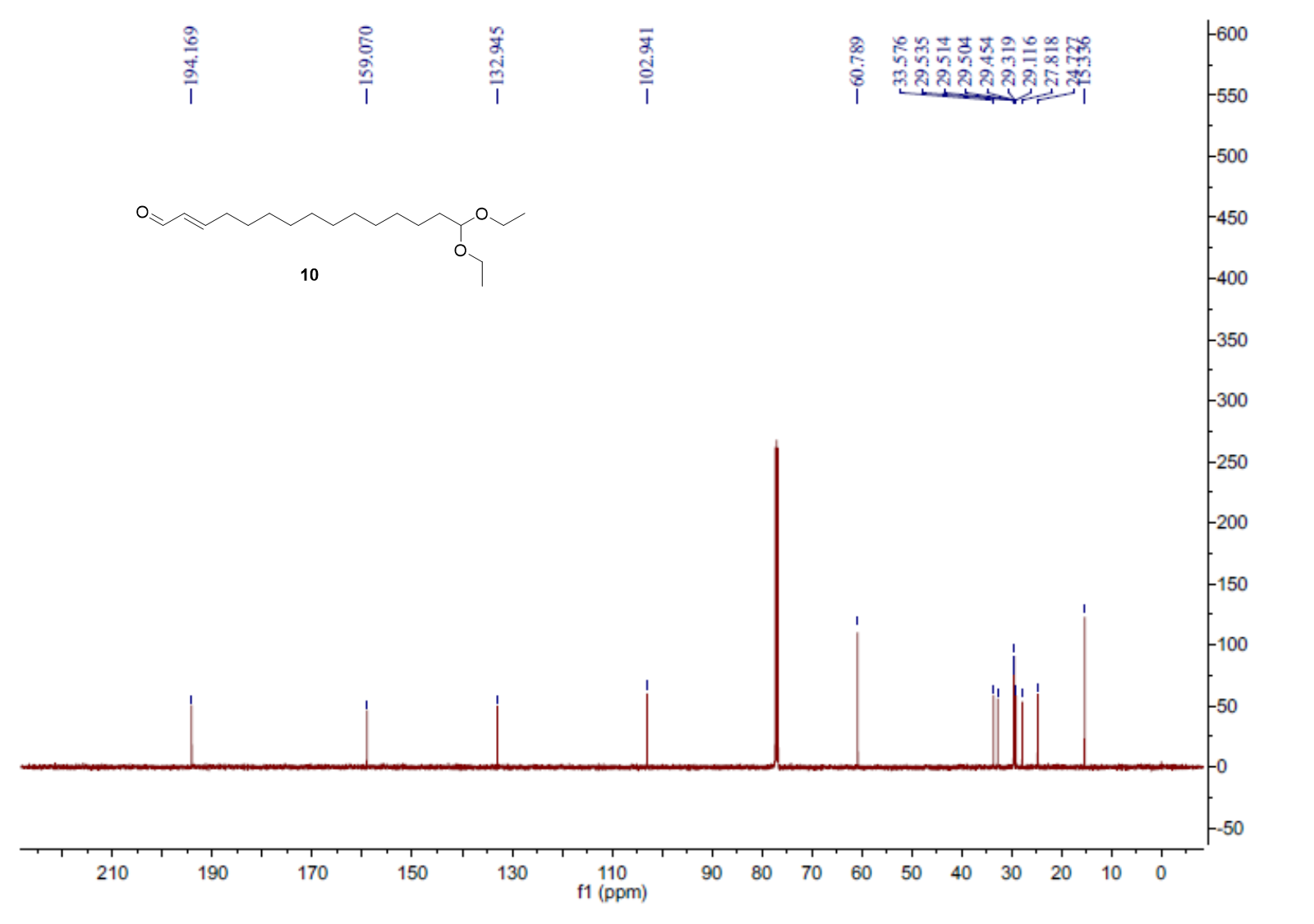


S1-16 The ^13^C NMR spectrum of compound **10**


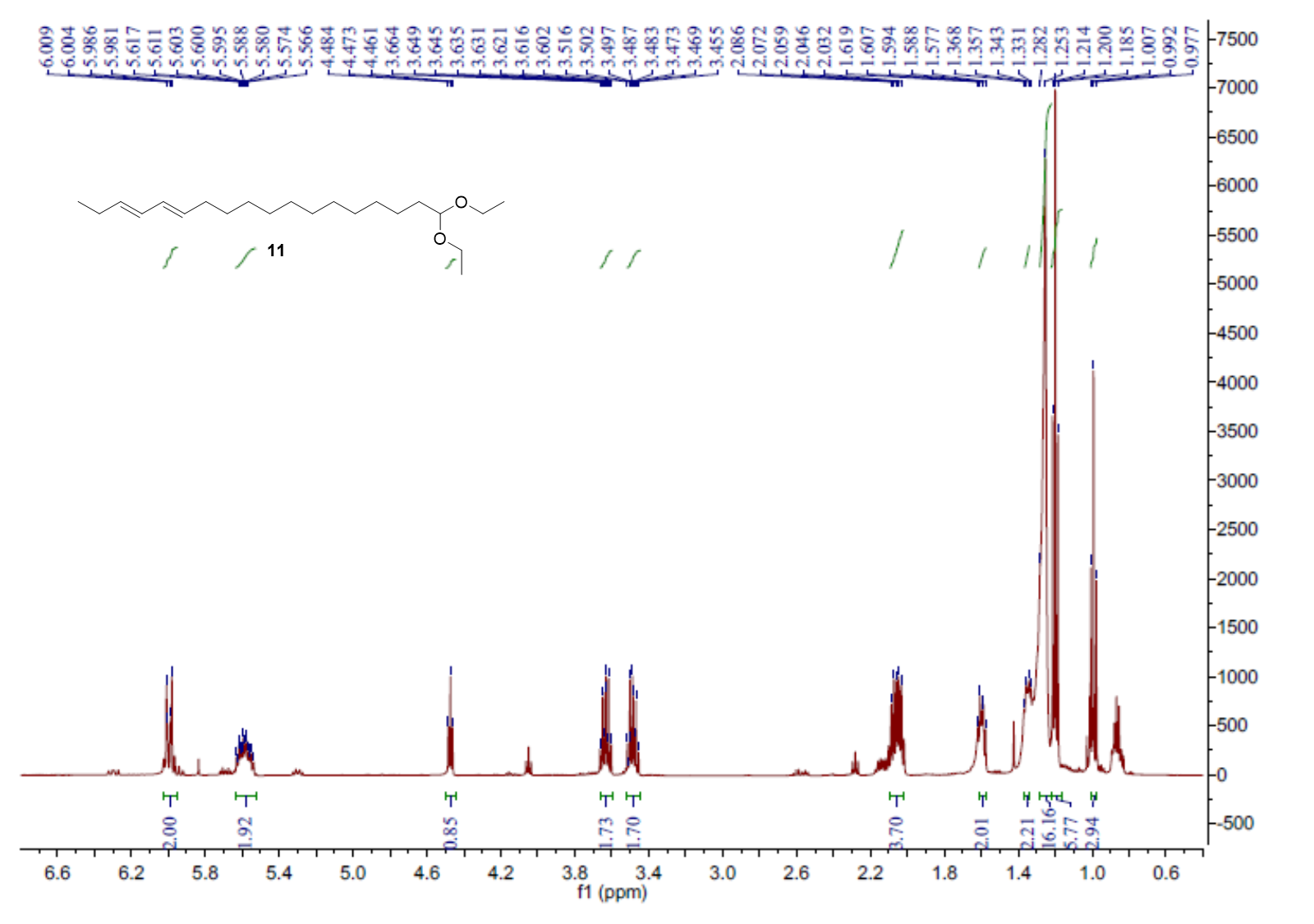


S1-17 The ^1^H NMR spectrum of compound **11**


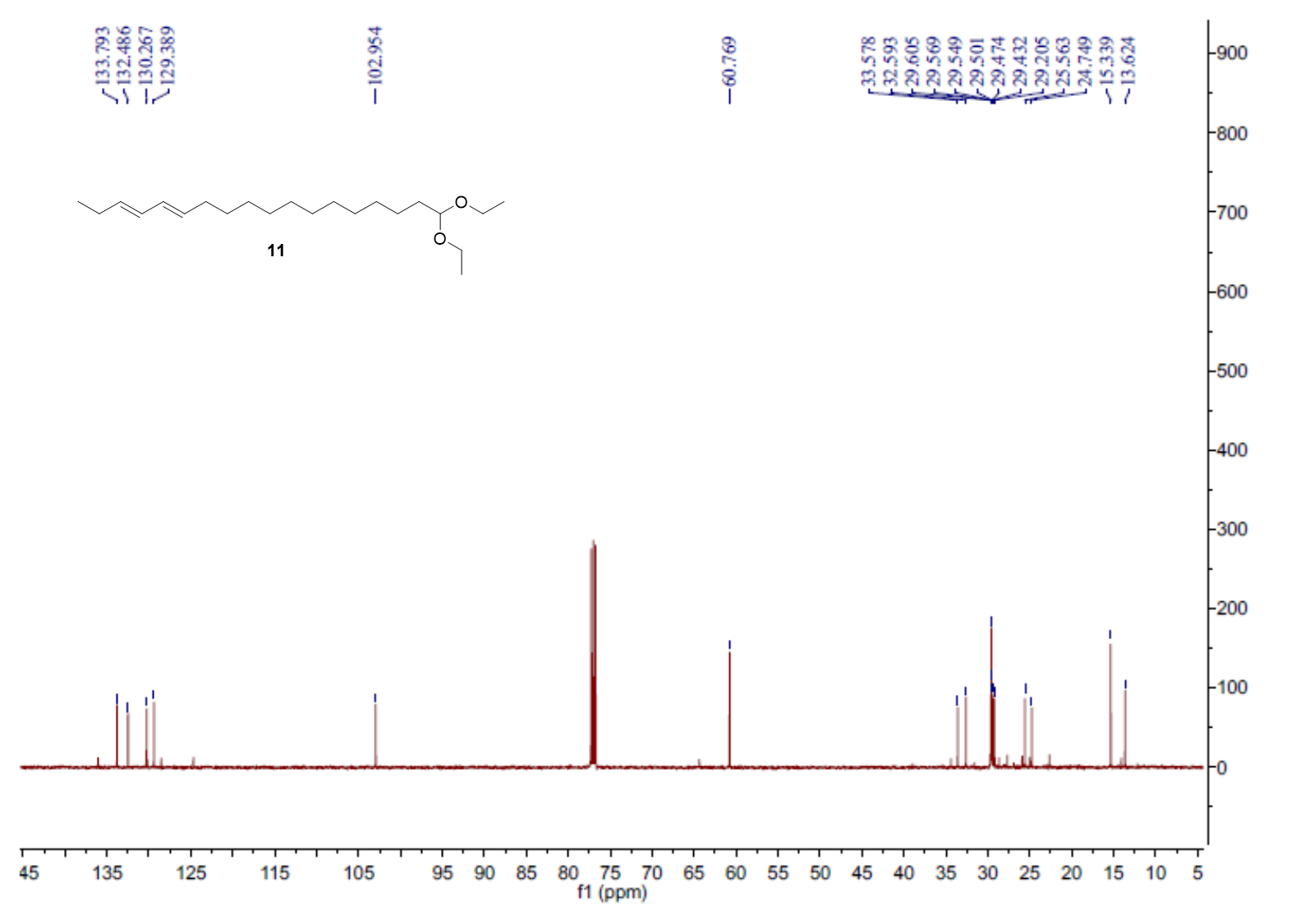


S1-18 The ^13^C NMR spectrum of compound **11**


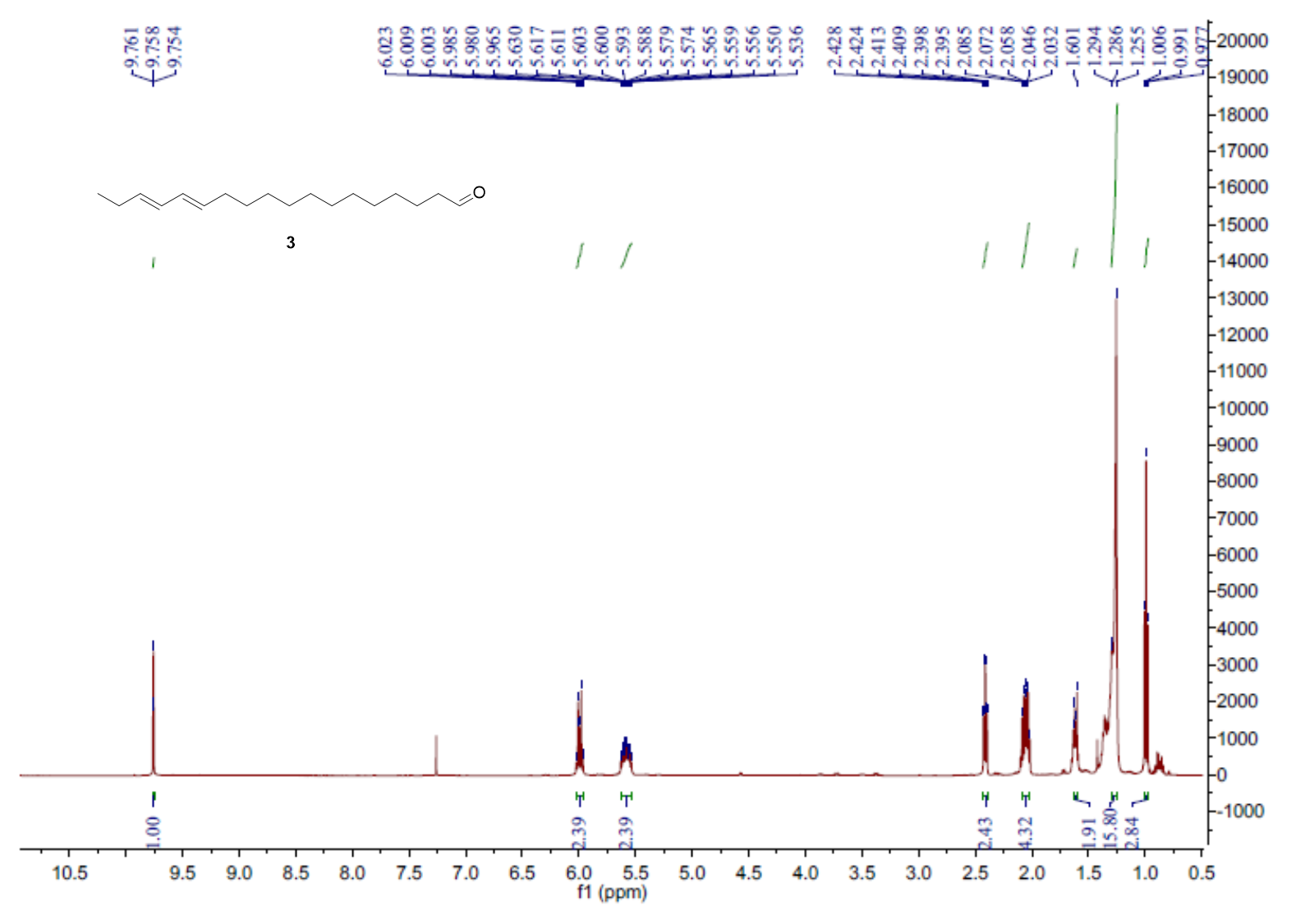
 S1-19 The ^1^H NMR spectrum of compound **3**


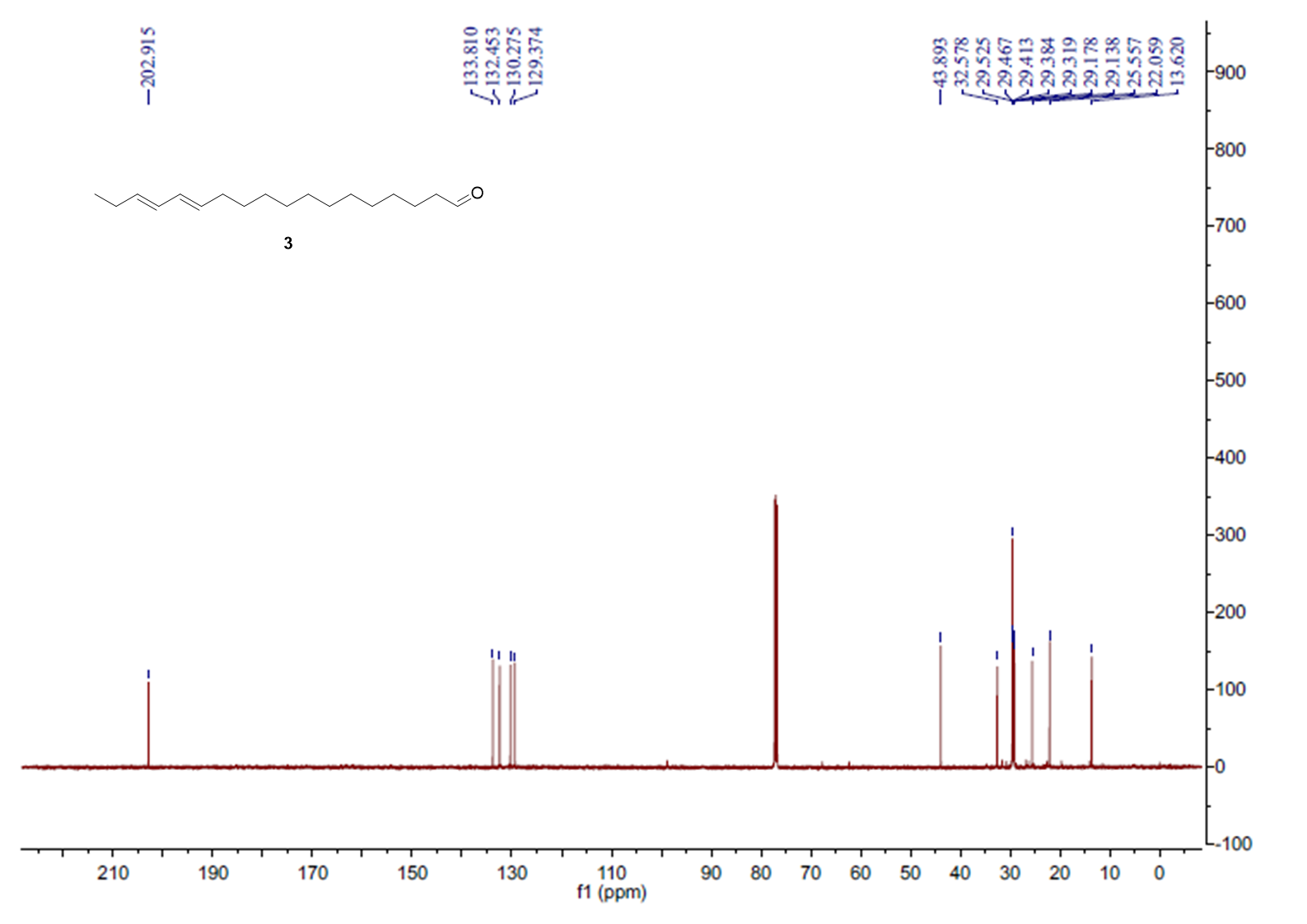


S1-20 The ^13^C NMR spectrum of compound **3**


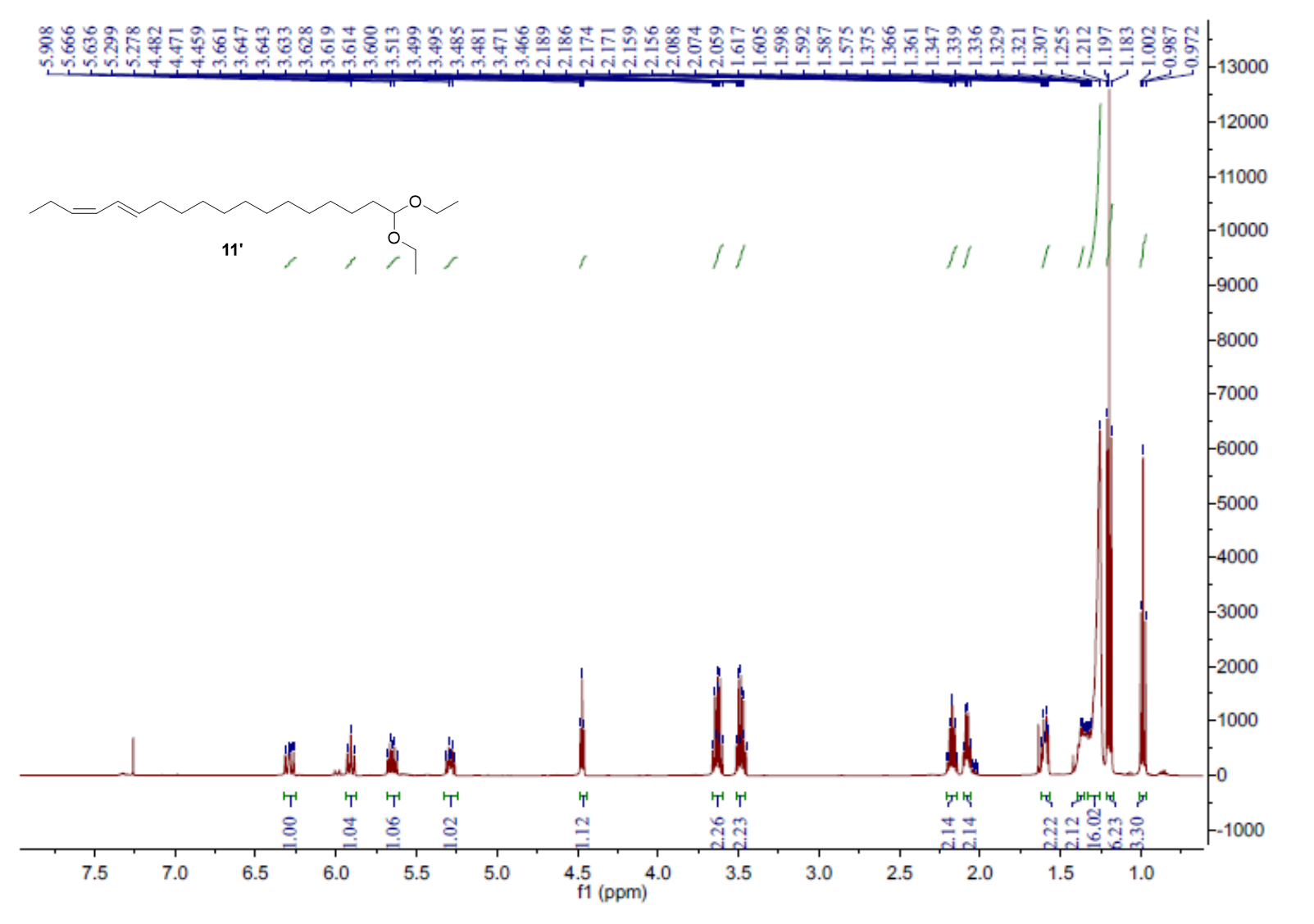


S1-21 The ^1^H NMR spectrum of compound **11′**


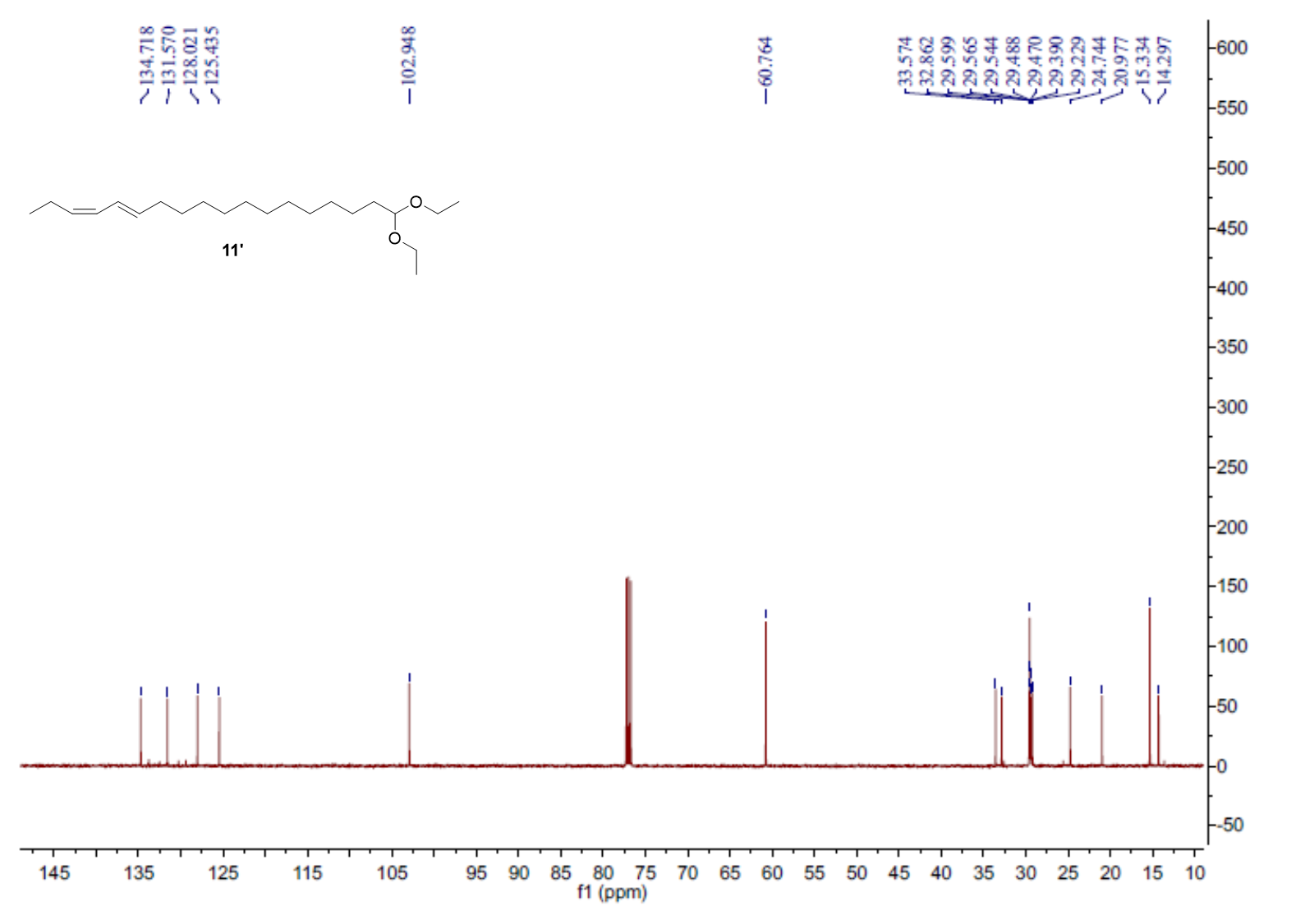


S1-22 The ^13^C NMR spectrum of compound **11′**


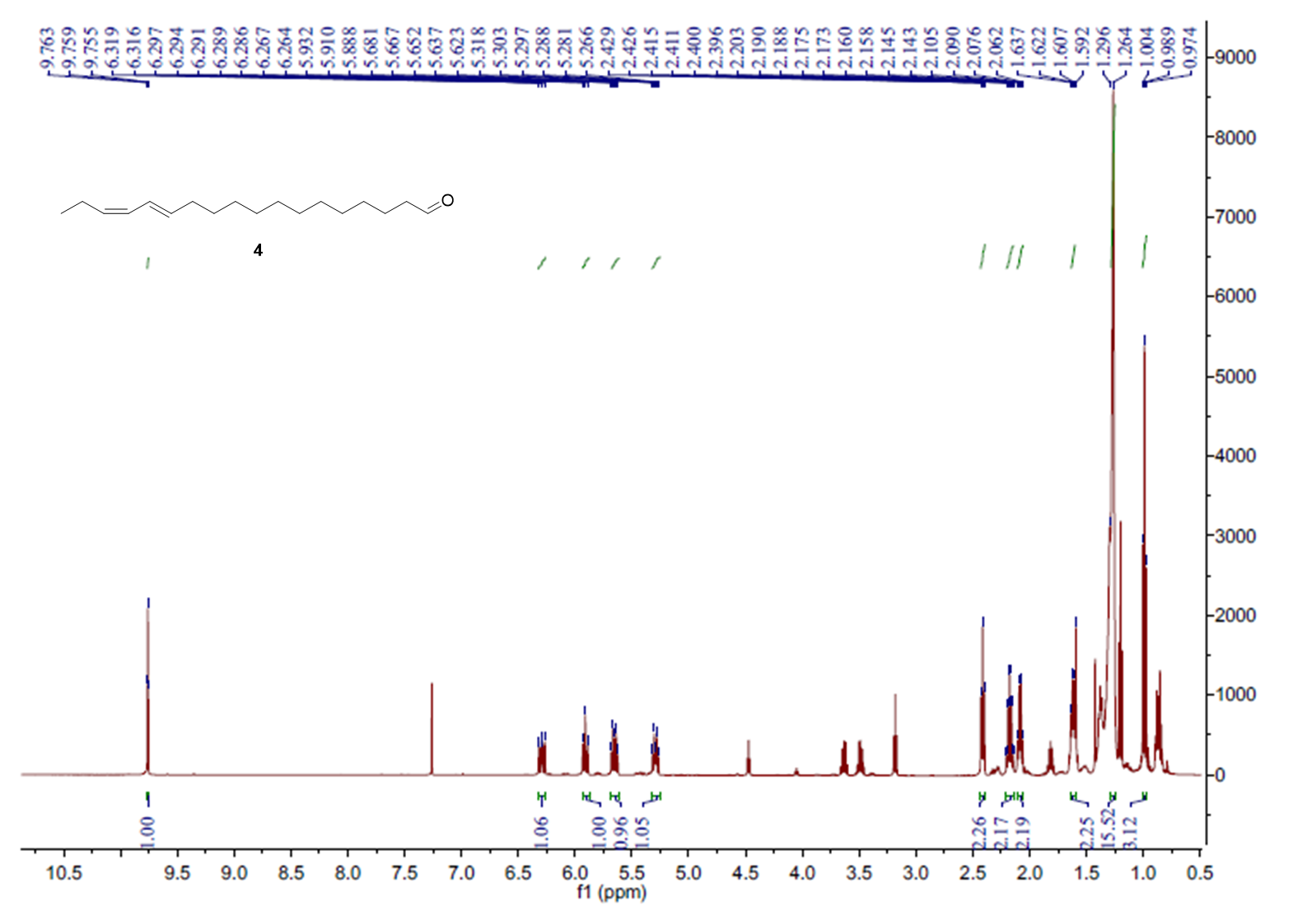


S1-23 The ^1^H NMR spectrum of compound **4**


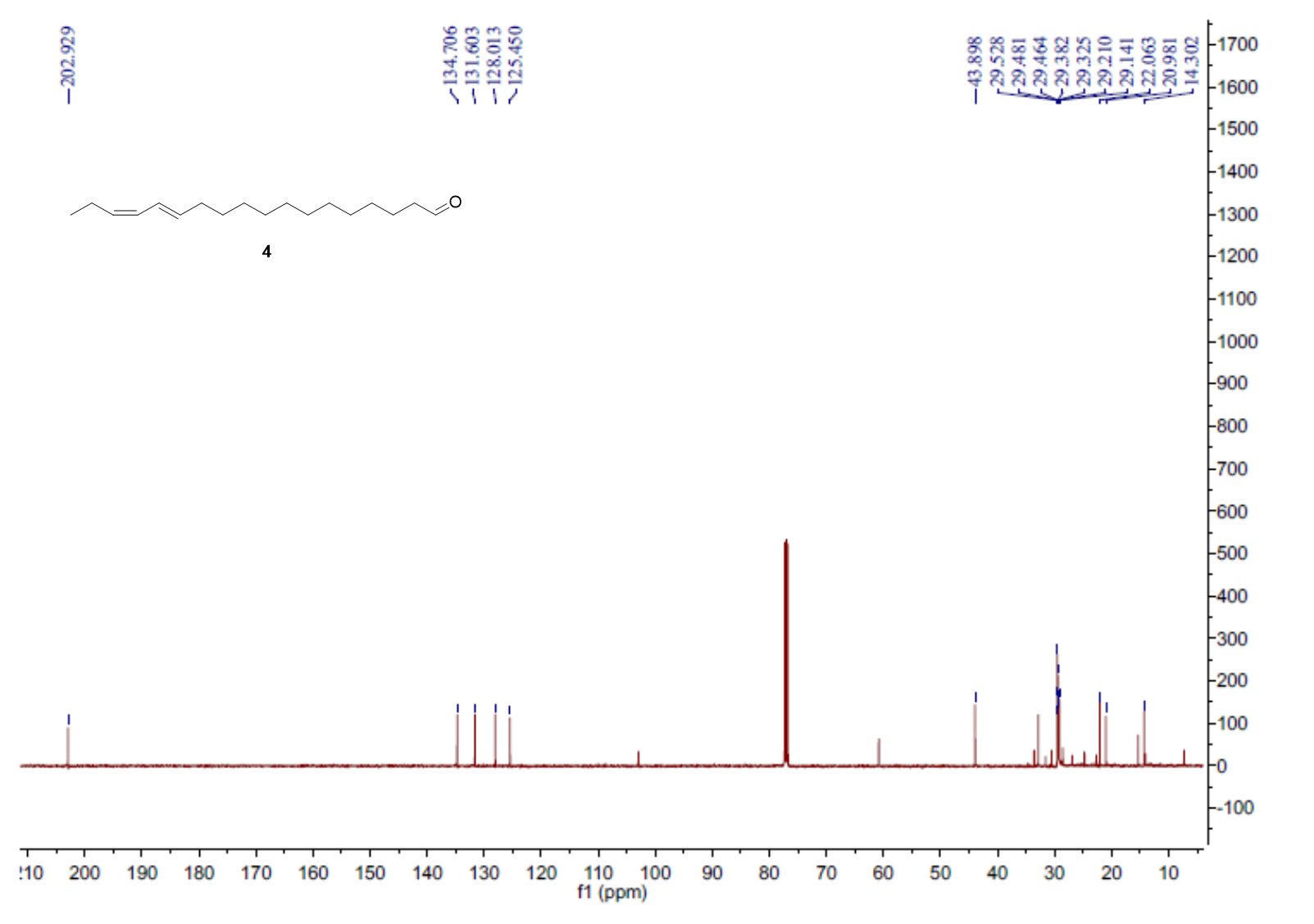


S1-24 The ^13^C NMR spectrum of compound **4**

**EI Mass spectra of the four isomers of 13,15-octadecadienal**


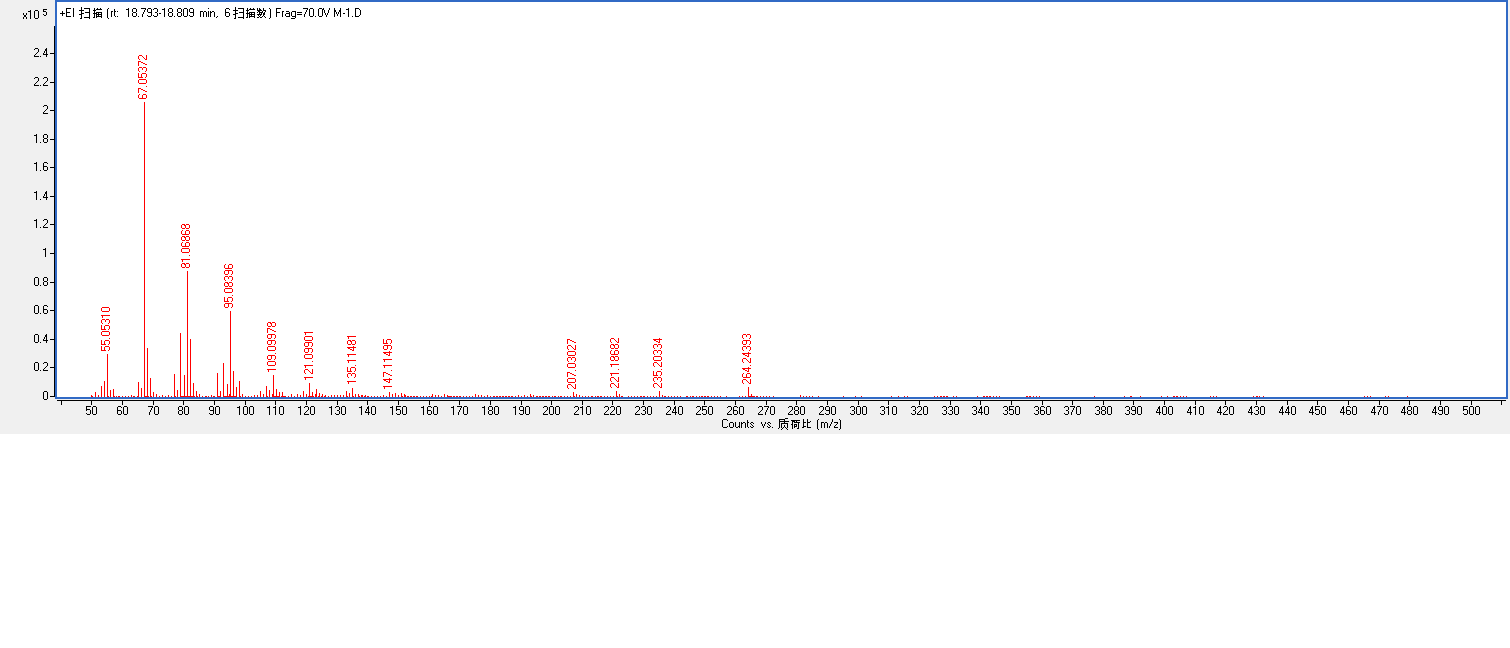


S1-25 EI Mass spectra of compound **1**


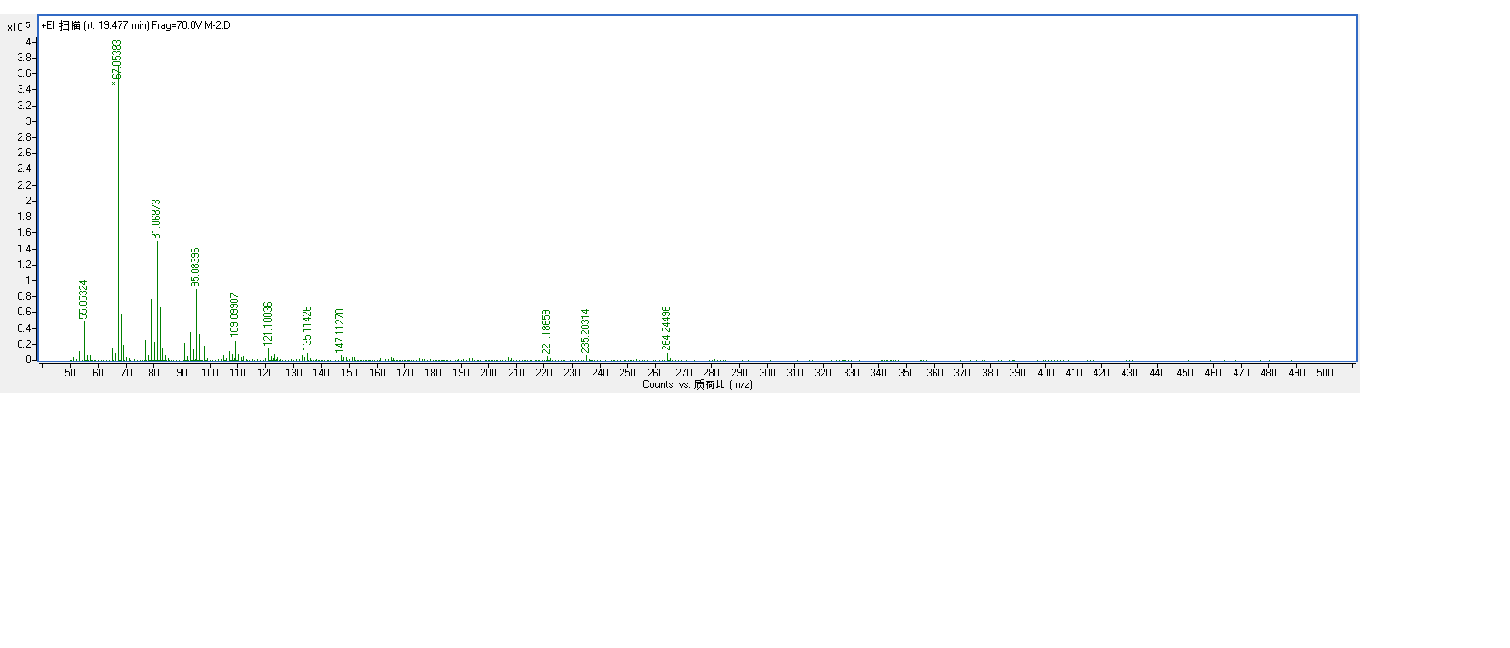


S1-26 EI Mass spectra of compound **2**


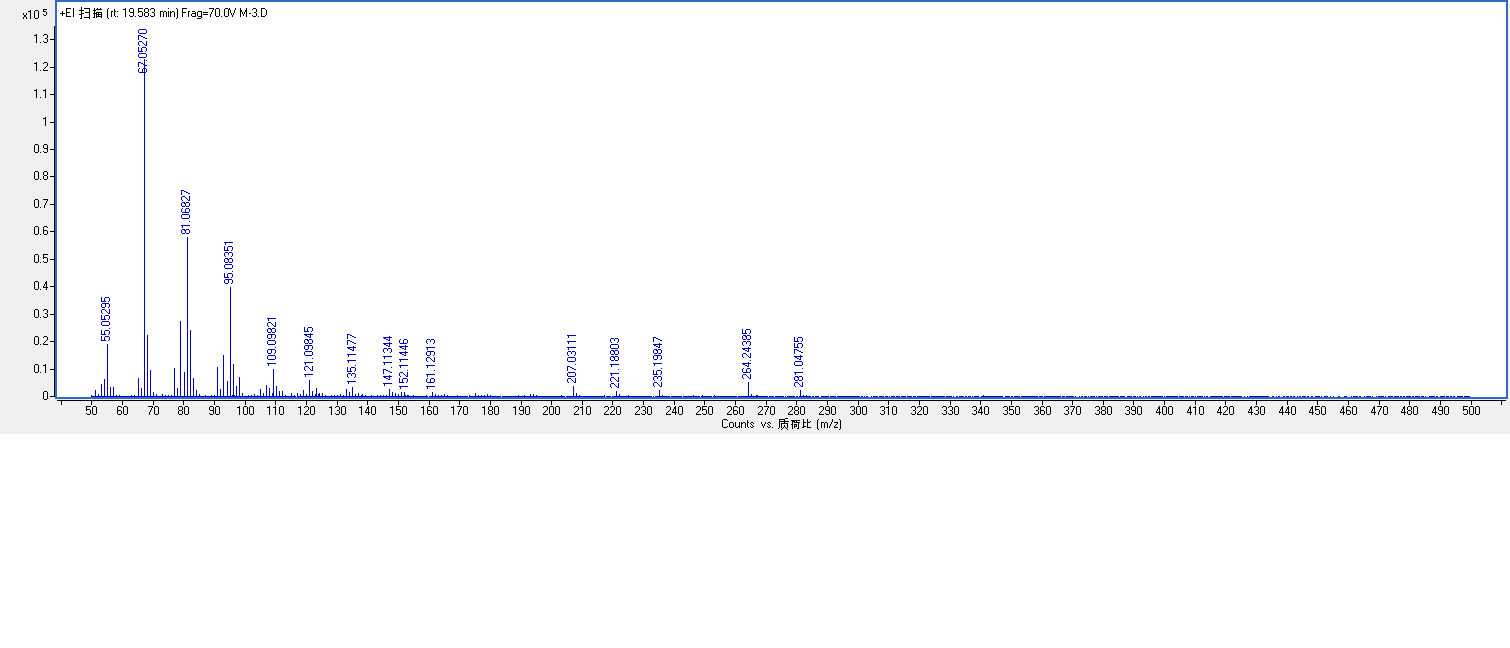


S1-27 EI Mass spectra of compound **3**


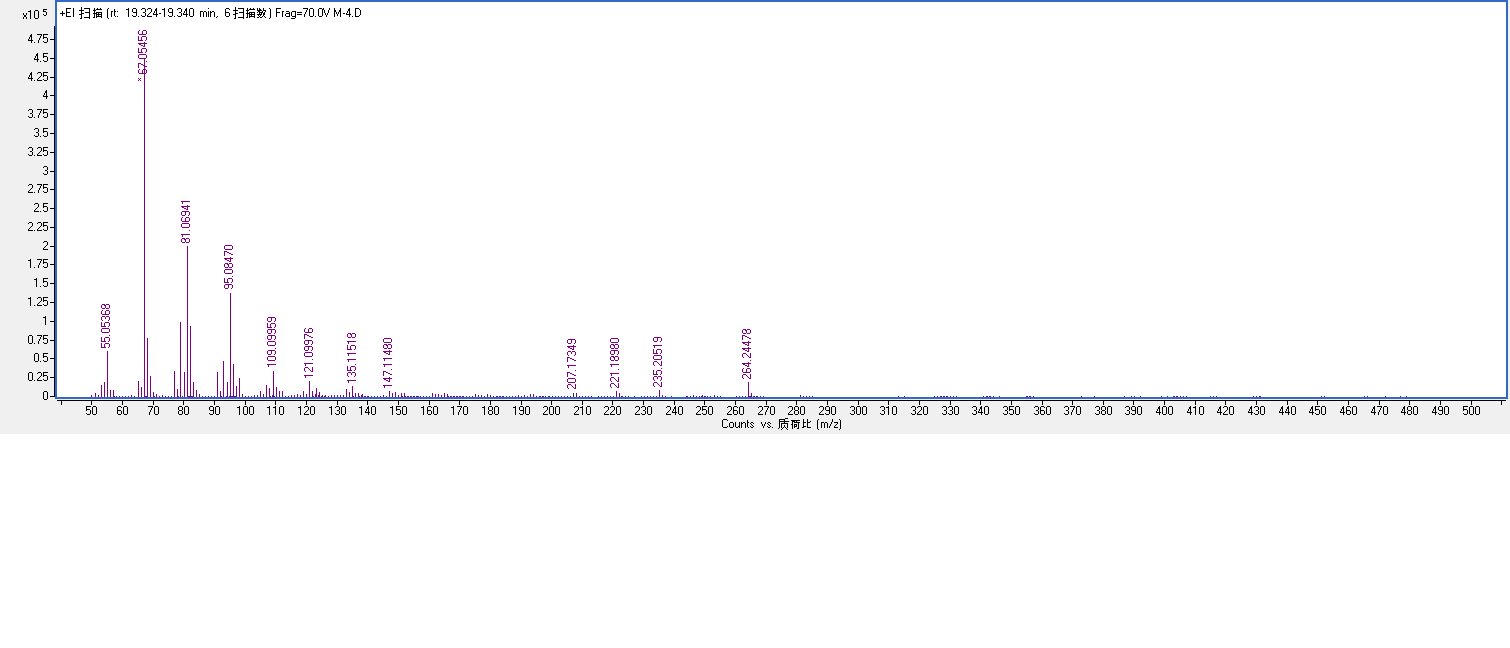


S1-28 EI Mass spectra of compound **4**
